# MoDnm1 Dynamin Mediating Peroxisomal and Mitochondrial Fission in Complex with MoFis1 and MoMdv1 Is Important for Development of Functional Appressorium in *Magnaporthe oryzae*

**DOI:** 10.1371/journal.ppat.1005823

**Published:** 2016-08-24

**Authors:** Kaili Zhong, Xiao Li, Xinyi Le, Xiangyi Kong, Haifeng Zhang, Xiaobo Zheng, Ping Wang, Zhengguang Zhang

**Affiliations:** 1 Department of Plant Pathology, College of Plant Protection, Nanjing Agricultural University and Key Laboratory of Integrated Management of Crop Diseases and Pests, Ministry of Education, Nanjing, China; 2 Department of Pediatrics, Louisiana State University Health Sciences Center, New Orleans, Louisiana, United States of America; Wageningen University, NETHERLANDS

## Abstract

Dynamins are large superfamily GTPase proteins that are involved in various cellular processes including budding of transport vesicles, division of organelles, cytokinesis, and pathogen resistance. Here, we characterized several dynamin-related proteins from the rice blast fungus *Magnaporthe oryzae* and found that MoDnm1 is required for normal functions, including vegetative growth, conidiogenesis, and full pathogenicity. In addition, we found that MoDnm1 co-localizes with peroxisomes and mitochondria, which is consistent with the conserved role of dynamin proteins. Importantly, MoDnm1-dependent peroxisomal and mitochondrial fission involves functions of mitochondrial fission protein MoFis1 and WD-40 repeat protein MoMdv1. These two proteins display similar cellular functions and subcellular localizations as MoDnm1, and are also required for full pathogenicity. Further studies showed that MoDnm1, MoFis1 and MoMdv1 are in complex to regulate not only peroxisomal and mitochondrial fission, pexophagy and mitophagy progression, but also appressorium function and host penetration. In summary, our studies provide new insights into how MoDnm1 interacts with its partner proteins to mediate peroxisomal and mitochondrial functions and how such regulatory events may link to differentiation and pathogenicity in the rice blast fungus.

## Author Summary

Dynamin superfamily members are involved in budding of transport vesicles and division of organelles in eukaryotic cells. To further understand how dynamins function in phytopathogenic fungi, we characterized several dynamin-related proteins from the rice blast fungus *M*. *oryzae*. In addition to revealing major conserved dynamin functions, we described how MoDnm1 interacts with mitochondrial fission protein MoFis1 and WD repeat adaptor protein MoMdv1 to mediate peroxisomal and mitochondrial fission, pexophagy and mitophagy. Importantly, we provided evidence to demonstrate that MoDnm1-, MoFis1- and MoMdv1-dependent peroxisomal and mitochondrial functions are linked to differentiation and pathogenicity of the rice blast fungus.

## Introduction

Dynamins are large GTPase superfamily proteins that are involved in scission (cleavage of the vesicle from the parent membrane) of nascent vesicles from parent membranes in eukaryotic cells [1]. Dynamins interact directly with the lipid bilayer at the necks of clathrin-coated pits to sever and release coated vesicles [2–5]. Dynamins contain five domains, including GTPase domain, middle domain, PH domain, GTPase effector domain (GED), and proline rich domain (PRD), while the dynamin-related proteins (DRPs) lack one or more of these domains or have additional domains. Dynamins and DRPs participate in a wide variety of cellular processes, including budding mitochondrial fission (mammalian Dlp1 and *Saccharomyces cerevisiae* Dnm1) and fusion (mammalian OPA1, *S*. *cerevisiae* Mgm1 and *Schizosaccharomyces pombe* Msp1), vacuolar fission (*S*. *cerevisiae* Vps1), interferon-induced anti-viral protection (fish Mx proteins), plant cell cytokinesis and membrane fission (*Arabidopsis thaliana* DRP proteins), as well as pathogen resistance [1, 6].

Peroxisomes are ubiquitous organelles that participate in a variety of important catabolic and anabolic processes, including reduction of hydrogen peroxide and lipid metabolism [7]. Peroxisomal oxidation, biogenesis and matrix protein importing are also important in cellular growth and differentiation [8–12]. Among two groups of peroxisomal proteins that have a pronounced influence on peroxisome size and abundance, DRPs are required for the scission of peroxisomal membranes [13], while Pex11-type peroxisome proliferators are involved in the proliferation of peroxisomes [14–16]. In *S*. *cerevisiae*, peroxisomal division depends on Fis1 and WD40 domain-containing adaptor proteins Mdv1 and Caf4 that recruit Dnm1 to the peroxisomal membrane [17, 18]. The DRP involvement in peroxisomal fission has also been found in plants. In *A*. *thaliana* DRP3A mutants, peroxisomes are elongated and reduced in number with aberrant mitochondria in contrast to the wild type plant [19].

Mitochondria are ubiquitous subcellular organelles essential to cellular physiology, energy supplies, amino acid biogenesis, certain metabolites, and programmed cell death. Mitochondria are dynamic organelles undergoing constant fusion and fission during cell division [20]. The equilibrium between fission and fusion is controlled by the activity of conserved molecular machines driven by self-assembling GTPases and DRPs [21]. Present evidence indicates that dynamin Dnm1 is the master regulator of mitochondrial division. Dnm1 self-assembles and exists at steady state in punctate structures in association with the outer mitochondrial membrane, often at points of membrane constriction and fission. In the budding yeast, four subunits of mitochondrial fission complex are identified as Dnm1, Fis1, Mdv1, and Caf4 [22–24]. Dnm1 forms atypical helical filaments that first encircle, then constrict the membrane-anchored protein Fis1 [25–28]. Dnm1, mammalian Dlp1, and *Caenorhabditis elegans* Drp1 are also known to mediate mitochondrial fission [26, 29]. Interestingly, Fis1 is required for the proper assembly and activation of the fission-mediating complex for mitochondrial division [22, 30]. Fis1 is proposed to be anchored in the outer mitochondrial membrane with its N-terminal region exposed to the cytosol and a short C-terminal tail protruding into the mitochondrial inter-membrane space [22]. In mammals, Fis1 recruits Dlp1 to peroxisomes and mitochondria with the assistance of adaptor protein Mdv1 or Caf4 [31].

Peroxisome-related virulence has been reported in pathogenic fungi. For example, the peroxisome-related proteins Pex11A and Pex19 are required for full virulence in *M*. *oryzae* [14, 28]. A recent study showed that a peroxisome-related protein, Pef1, mediated peroxisomal fission during appressorium formation is important for infection of the rice blast fungus [32]. Mitochondria regulate virulence in *Heterobasidion annosum* [33] and the loss of mitochondrial function in *Candida glabrata* and *C*. *albicans* results in a defect in virulence [34–36]. The changes in mitochondrial morphology toward more tubular-structured organelles play a positive role in virulence in *Cryptococcus gattii* [37]. Another study showed that reduced virulence associated with dysfunctional mitochondria is probably due to reduced fitness, metabolic changes, and sensitivity to oxidative stress caused by defective respiration [38].

Autophagy is a common and evolutionarily conserved process where cytosol and organelles can be degraded and recycled in all eukaryote cells [39–41]. Autophagy not only recycles intracellular components to compensate for nutrient deprivation but also selectively eliminates organelles to regulate their number and maintain quality control [42]. It was first identified by electron microscopy and considered as nonselective for its cytosolic cargos [43]. Later analyses identified different types of selective autophagy, including glycogen autophagy [44], mitophagy [45] and pexophagy [46]. A set of evolutionarily conserved autophagy-related genes (*ATG* genes) were initially identified in yeast [47, 48]. The scaffold protein Atg11 and the pexophagy receptor Atg36 interact with both Dnm1 and Vps1, which occurs in mitochondria and peroxisomes [49]. In *M*. *oryzae*, a total of 22 *ATG* genes were identified, with *MoATG8* and *MoATG24* being implemented in autophagy induction and glycogen autophagy during conidiogenesis, respectively [50].

Although relationships and functions among Dnm1, Mdv1 and Fis1 were explored in *S*. *cerevisiae* and mammals, they were less understood in filamentous and pathogenic fungi, including *M*. *oryzae*. Recent studies have showed that PEX proteins such as Pex11A and Pex19 are required for peroxisomal proliferation and virulence in *M*. *oryzae* [14, 28]. Here, we described MoDnm1 function in peroxisomal and mitochondrial fission and in the appressorium-mediate infection of the rice blast fungus. We also described that MoDnm1-mediated peroxisomal and mitochondrial fission involves conserved functions from mitochondrial fission protein MoFis1 and WD40 domain-containing adaptor protein MoMdv1.

## Results

### *M*. *oryzae* encodes at least four dynamin-related proteins

**Fig 1:**
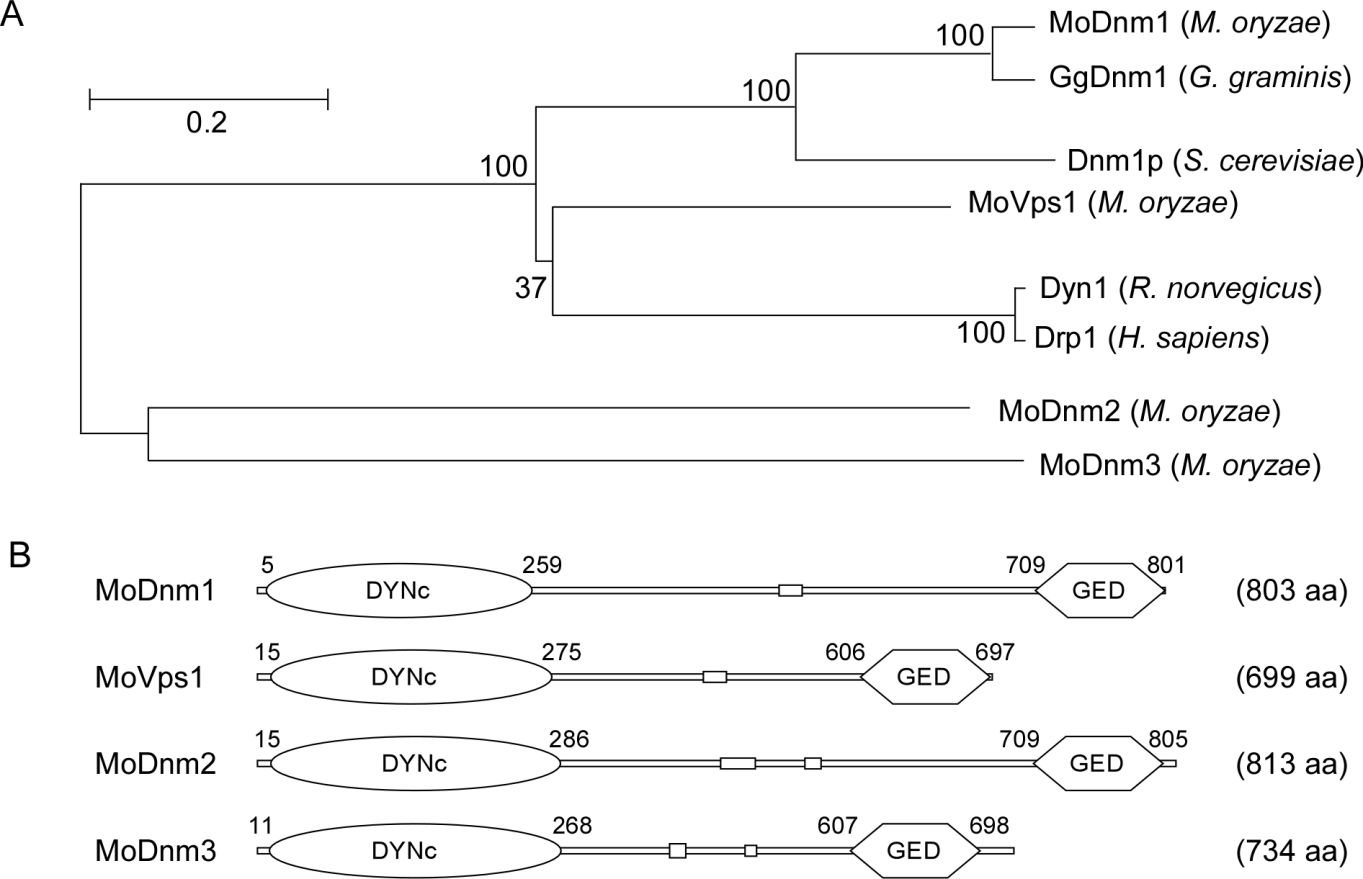
Homology prediction and phylogenetic analysis of dynamin-related proteins in *M*. *oryzae*. (A) Phylogenetic trees of dynamin-related proteins from several organisms were constructed using the CLUSTAL_W and MEGA 5.1 programs by the neighbour-joining method with 1000 bootstrap replicates. Species names and GenBank accession numbers are as follows: XP_003717217.1 (*M*. *oryzae* MoDnm1); XP_003712225.1 (*M*. *oryzae* MoVps1); XP_003708884.1 (*M*. *oryzae* MoDnm2); XP_003721138.1 (*M*. *oryzae* MoDnm3); XP_009217281.1 (*G*. *graminis* GgDnm1); NP_013100.1 (*S*. *cerevisiae* Dnm1p); NP_542420.1 (*R*. *norvegicus* Dyn1); XP_011516636.1 (*H*. *sapiens* Drp1). (B) Schematic representation of dynamin-related proteins. DYNc, large GTPase domain; GED, GTPase effector domain; aa, amino acids.

Since dynamin proteins are highly conserved, we searched the available genomes of *M*. *oryzae* (http://www.broadinstitute.org/annotation/genome/magnaporthe_comparative/MultiHome.html) by BLAST using the *S*. *cerevisiae* dynamin Dnm1 protein [1] as the reference [51] and identified the MGG_06361 genetic loci encoding the Dnm1 homolog MoDnm1. Since previous studies indicated that DRPs are distinct from other GTPases by having a large GTPase domain (~300 amino acids), a middle domain, and a GED domain [1], we identified three DRP candidates: MoVps1 (MGG_09517), MoDnm2 (MGG_02114), and MoDnm3 (MGG_02648). Sequence analysis revealed that MoDnm1 is also homologous to *Gaeumannomyces graminis* GgDnm1, MoVps1 shares a high sequence conservation with *Rattus norvegicus* Dyn1 and *Homo sapiens* Drp1, MoDnm2 and MoDnm3 are exclusive to *M*. *oryzae* (Fig 1A), and all four *M*. *oryzae* proteins contain large GTPase and GED domains (Fig 1B). These results suggest that dynamin family members of *M*. *oryzae* are conserved with many other organisms, with Dnm1 being the most conserved dynamin protein.

### MoDnm1 is important for vegetative growth, conidiation, and virulence

To examine functions of dynamin proteins in *M*. *oryzae*, we generated deletion mutant specific to each dynamin-related gene. For reasons unknown, a Δ*Movps1* (MGG_09517) mutant could not be obtained despite screening of more than three thousand transformants. This may indicate that MoVps1 plays an essential role in *M*. *oryzae*. For the other three dynamin-related genes (MGG_06361, MGG_02114 and MGG_02648), deletion mutants were generated (S1A and S1B Fig) and examined for phenotypic changes in vegetative growth and conidiation. The Δ*Modnm1* mutant displayed significantly attenuated growth in either the complete medium (CM) or minimal medium (MM), and showed marked reduction in conidiation in comparison to the wild type strain (Guy11). No phenotypic changes were found in the Δ*Modnm2* and Δ*Modnm3* mutants (S1 Table). These results indicate that MoDnm1, but not MoDnm2 and MoDnm3, is involved in vegetative growth and conidiation.

**Fig 2:**
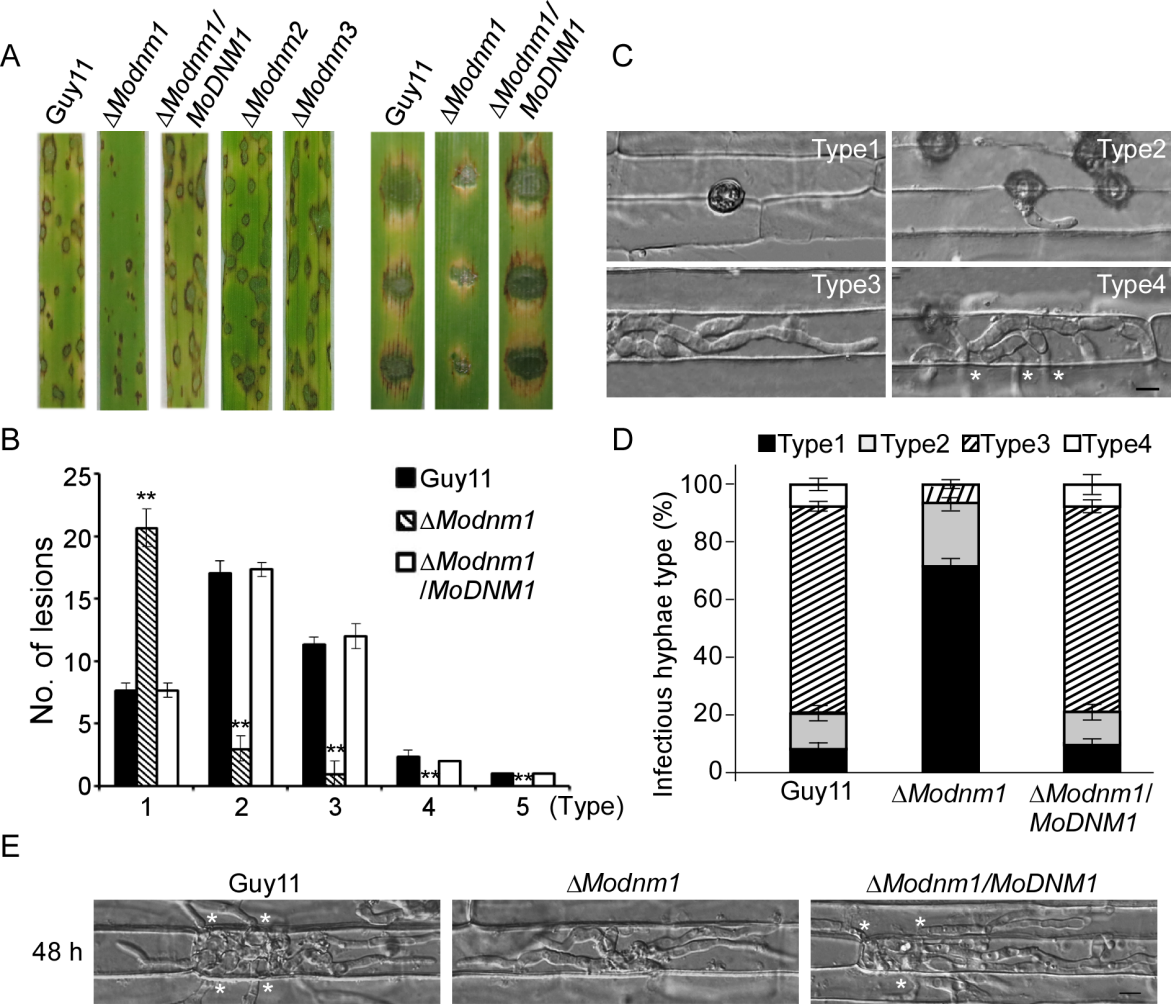
MoDnm1 is important for full virulence of *M*. *oryzae*. (A) Rice (*Oryza sativa* cv. CO39) seedlings (left) were sprayed with conidial suspensions and examined 7 dpi. Detached barley (*Hordeum vulgare* cv. Four-arris) (right) leaves were drop-inoculated with conidial suspensions and examined 5 dpi. (B) Quantification of lesion types (0, no lesion; 1, pinhead-sized brown specks; 2, 1.5 mm brown spots; 3, 2–3 mm grey spots with brown margins; 4, many elliptical grey spots longer than 3 mm; 5, coalesced lesions infecting 50% or more of the leaf area). Lesions were measured at 7 dpi, numbers within an area of 1.5 cm^2^ were counted, and experiments were repeated three times with similar results. Asterisks represent significant differences (Duncan's new multiple range test, *p*<0.01). (C, D) Detailed observation and statistical analysis of invasive growth in rice sheath cells at 36 hpi. For each sample, appressorium penetration sites (n = 100) were checked and the invasive hyphae (IH) were rated from type 1 to 4. Error bars represent ±SD from three independent experiments. Bar = 5 μm. Asterisks indicate IH extended to surrounding cells. (E) Comparative analysis of invasive hyphal growth of the wild type, Δ*Modnm1* mutant and complemented strains in rice sheath cells at 48 hpi. Asterisks indicate IH extended to surrounding cells.

To examine the role of dynamins in fungal pathogenicity, we inoculated conidial suspensions of the wild type, Δ*Modnm1*, Δ*Modnm2*, Δ*Modnm3* mutants and complemented strains on the susceptible rice cultivar CO-39. The Δ*Modnm1* mutant produced small, restricted lesions, in contrast to Δ*Modnm2* and Δ*Modnm3* mutants that were as virulent as the wild type at 7 days post-inoculation (dpi). The similar result was also obtained on the detached barley cultivar Four-arris leaves (Fig 2A). Disease lesions on rice leaves were also quantified by a ‘lesion-type’ scoring assay [52]. The Δ*Modnm1* mutant yielded more restricted small dark brown spot lesions (type 1) and less type 2 to 5 lesions, in comparison to the wild type and complemented strains (Fig 2B). To further elaborate these observations, we examined penetration and invasive hyphal (IH) growth in rice sheath cells. The wild type strain showed at least a rate of 90% successful appressorium penetration events, with more than 80% of type 3 (extend but limit in one cell) and 4 (extend to surrounding cells) infectious hyphae at 36 hours post-inoculation (hpi). In contrast, only 32% of Δ*Modnm1* appressoria had successful penetration with less than 18% of penetration sites showing type 3 and 4 invasive growth (Fig 2C and 2D). Moreover, Δ*Modnm1* mutant invasive hyphae were limited within one cell at 48 hpi, whereas wild type invasive hyphae extended to surrounding cells (Fig 2E). All these results indicate that MoDnm1 plays an important role in invasive hyphal growth and host colonization.

### MoMdv1 functions as an adaptor protein linking MoDnm1 to MoFis1

**Fig 3:**
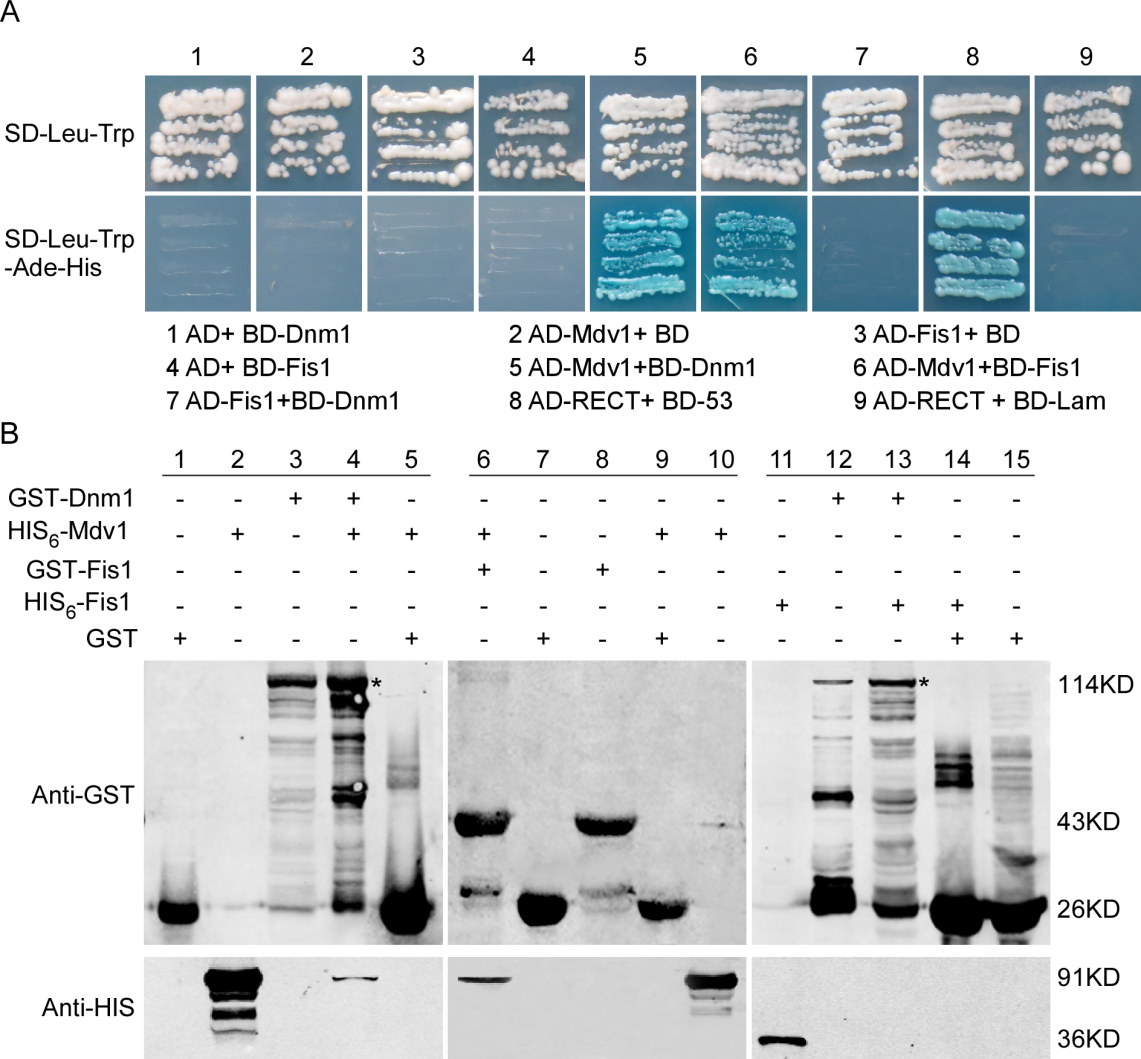
MoMdv1 functions as an adaptor linking MoDnm1 to MoFis1. (A) Yeast two hybrid assays for the interaction between MoDnm1, MoFis1 and MoMdv1. The AD and BD plasmids were co-transformed into yeast AH109, and transformants were plated on SD-Leu-Trp for 3 d and on selective SD-Leu-Trp-His-Ade with 1 mM X-gal and 5 mM 3-AT (3-amino-1,2,4-triazole) for 5 d. (B) GST pull down assays for MoDnm1, MoFis1 and MoMdv1. His_6_-Mdv1, His_6_-Fis1, GST-Dnm1 and GST-Fis1 were expressed and purified by affinity chromatography. Bound proteins were separated by SDS-PAGE in duplicate and analyzed by Western blotting with monoclonal anti-His (Mouse; M20001; Abmart) and anti-GST antibodies (Mouse; M20007; Abmart). Asterisks indicate GST-Dnm1.

In the budding yeast and mammals, Dnm1 functions in peroxisomal and mitochondrial fission through a complex with Fis1 and Mdv1 proteins [24, 26, 29–31, 53]. To test whether MoDnm1 functions similarly, we identified *M*. *oryzae* MGG_06075 (MoFis1) and MGG_01711 (MoMdv1) as yeast Fis1 and Mdv1 homologs, respectively, and tested their interactions through yeast two-hybrid screen. MoDnm1 and MoMdv1, and MoMdv1 and MoFis1 were respectively co-transformed into the yeast host, and selected on SD-Leu-Trp-His-Ade medium containing 1 mM X-gal and 5 mM 3-AT (3-amino-1,2,4-triazole). MoMdv1 was found to interact with both MoDnm1 and MoFis1 (Fig 3A). These interactions were further validated by the protein pull down assay in which GST-Dnm1 and GST-Fis1 were bound to His_6_-Mdv1 (Fig 3B). Interestingly, a direct interaction cannot be established between MoDnm1 and MoFis1 as GST-Dnm1 could not bound to His_6_-Fis1, suggesting that MoDnm1 does not directly couple to MoFis1 and MoMdv1 functions as an adaptor linking MoDnm1 with MoFis1.

### MoMdv1 and MoFis1 are important for conidiation and pathogenicity

To examine functions of MoFis1 and MoMdv1, we also generated respective mutant strains (S1C Fig). Similar to the Δ*Modnm1* mutant, Δ*Mofis1* and Δ*Momdv1* mutants were significantly attenuated in growth and conidiation, with normal conidium germination and appressorium formation, when compared with the wild type and complemented strains (Table 1). These results indicate that MoFis1 and MoMdv1 share similar functions with MoDnm1 in the regulation of vegetative growth and conidial formation.

**Table 1:** Comparison of mycological characters among Guy11 and deletion mutant strains.

Strain	Growth (cm)^a^				Conidiation (× 10^4^/cm^2^)^b^	Germination rate (%)^c^	Appressorium formation (%)^d^
	CM	MM	OM	SDC			
Guy11	5.09±0.1	4.52±0.1	4.32±0.1	3.86±0.1	13.8±0.4	98.8.±1.0	99.0.±1.7
Δ*Modnm1*	3.61±0.1**	2.42±0.1**	3.57±0.1**	3.10±0.1**	3.8±0.4**	98.6±1.4	98.8±1.2
Δ*Mofis1*	3.53±0.2**	3.33±0.1**	3.04±0.1**	3.02±0.1**	8.9±0.6**	98.6±1.4	98.8±1.2
Δ*Momdv1*	3.28±0.1**	3.30±0.1**	2.81±0.1**	2.98±0.2**	9.6±0.8**	98.8±1.6	98.5±1.5
Δ*Modnm1*/*MoDNM1*	4.97±0.1	4.53±0.1	4.33±0.2	3.92±0.2	13.2±0.8	99.0.±1.2	98.7±1.4
Δ*Mofis1*/*MoFIS1*	4.98±0.1	4.43±0.1	4.35±0.1	3.78±0.2	13.0±0.8	99.1.±1.4	98.9±1.4
Δ*Momdv1/MoMDV1*	5.02±0.2	4.49±0.1	4.29±0.1	3.82±0.2	13.4±0.7	98.6.±1.2	99.1.±1.6

^a^ Colony diameter was measured after 7-day growth in 28°C.

^b^ Quantification of the conidial production of the indicated strains from SDC cultures.

^c^ Percentage of conidial germination on artificial surface at 4 hpi.

^d^ Percentage of appressorium formation on artificial surface at 24 hpi. ±SD was calculated from three repeated experiments and asterisks indicate statistically significant differences (Duncan's new multiple range test, ** means *p*<0.01).

**Fig 4:**
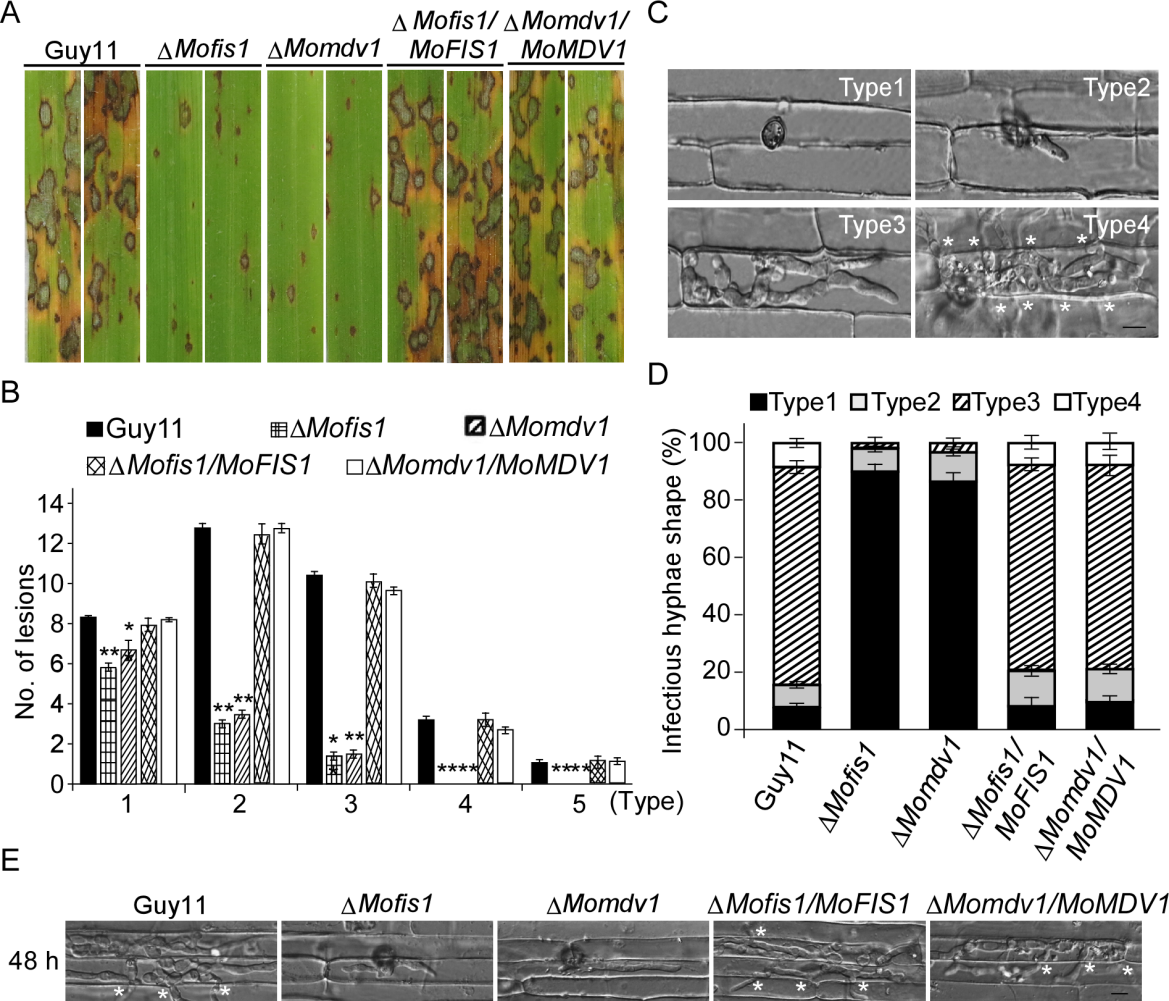
MoFis1 and MoMdv1 are important for virulence in *M*. *oryzae*. (A) Rice (*Oryza sativa* cv. CO39) seedlings were sprayed with conidial suspensions and examined 7 dpi. (B) Quantification of lesion types (per 1.5 cm^2^) on susceptible rice cultivar CO39 spayed with conidia of Guy11, Δ*Mofis1*, Δ*Momdv1*, Δ*Mofis1/MoFIS1*, and Δ*Momdv1/MoMDV1* strains. Asterisks represent significant differences (Duncan's new multiple range test, *p*<0.01). (C, D) Detailed observation and statistical analysis for infectious growth in rice sheath cells at 36 hpi. Appressorium penetration sites (n = 100) were observed and invasive hyphae were rated from type 1 to 4. Error bars represent ±SD from three independent experiments. Bar = 5 μm. Asterisks indicate IH extended to surrounding cells. (E) Invasive hyphal growth of the Δ*Mofis1* and Δ*Momdv1* mutants in rice sheath cells at 48 hpi. Asterisks indicate IH extended to surrounding cells.

We also assessed virulence of the Δ*Momdv1* and Δ*Mofis1* mutant on rice and found that both caused smaller and less lesions than the wild type strain (Fig 4A and 4B). To verify this finding, we also performed penetration and invasive hyphal growth assays in rice sheath and found that less than 11% and 14% of appressorium penetration was successful in Δ*Mofis1* and Δ*Momdv1* mutants, respectively. Moreover, less than 5% of penetration sites exhibited type 3 and 4 lesions by both mutants (Fig 4C and 4D). Limited infectious hyphae of Δ*Mofis1* and Δ*Momdv1* mutants were observed after 48 h inoculation, which is similar to the Δ*Modnm1* mutant (Fig 4E). Taken together, all these results indicate that MoMdv1 and MoFis1 play important roles in virulence by affecting host penetration and invasive hyphal growth.

### MoDnm1, MoMdv1, and MoFis1 regulate the appressorium turgor pressure

Appressorium-mediated penetration requires high internal turgor pressure to generate sufficient mechanical force to breach the rice leaf cuticle [10, 54, 55]. As appressorial formation appears to be normal in the Δ*Modnm1*, Δ*Mofis1*, and Δ*Momdv1* mutants, we examined whether the defect in turgor generation resulted in the reduction in pathogenicity. In incipient cytorrhysis (cell collapse) assay using the 1–4 molar concentration of glycerol solution, appressoria of the Δ*Modnm1*, Δ*Mofis1* and Δ*Momdv1* mutants showed higher collapse ratios in comparison to that of the wild type (S2 Fig). These results suggest that the reduced pathogenicity of the Δ*Modnm1*, Δ*Mofis1*, and Δ*Momdv1* mutants are due to the aberrant development of functional appressoria.

In *M*. *oryzae*, effective transfer of glycogen and triacylglycerol is required for appressorial maturation and appressorium-mediated host penetration [10, 55–57]. We therefore examined the cellular distribution of glycogen and lipid bodies during appressorium development. Upon iodine-staining abundant glycogen was seen in conidia, germ tubes, and appressoria. We found that mobilization of glycogen was retarded in the Δ*Modnm1* mutant with glycogen depletion in conidia until 12 h, in comparison to 6–8 h in the wild type (S3A Fig). Next, we investigated the distribution of lipid bodies by Nile red staining and found that there was no difference on intracellular lipid storage between the Δ*Modnm1* mutant and the wild type (S3B Fig). Additionally, no difference was found in the distribution of glycogen and lipid during appressorium morphogenesis between the Δ*Mofis1* and Δ*Momdv1* mutants, and the wild type strain (S3C and S3D Fig). These results suggest that the defects of these mutants in appressorium turgor pressure are not attributed to the mobilization of glycogen and lipid droplets mobilization.

### MoDnm1, MoMdv1 and MoFis1 are required for normal endocytosis

**Fig 5:**
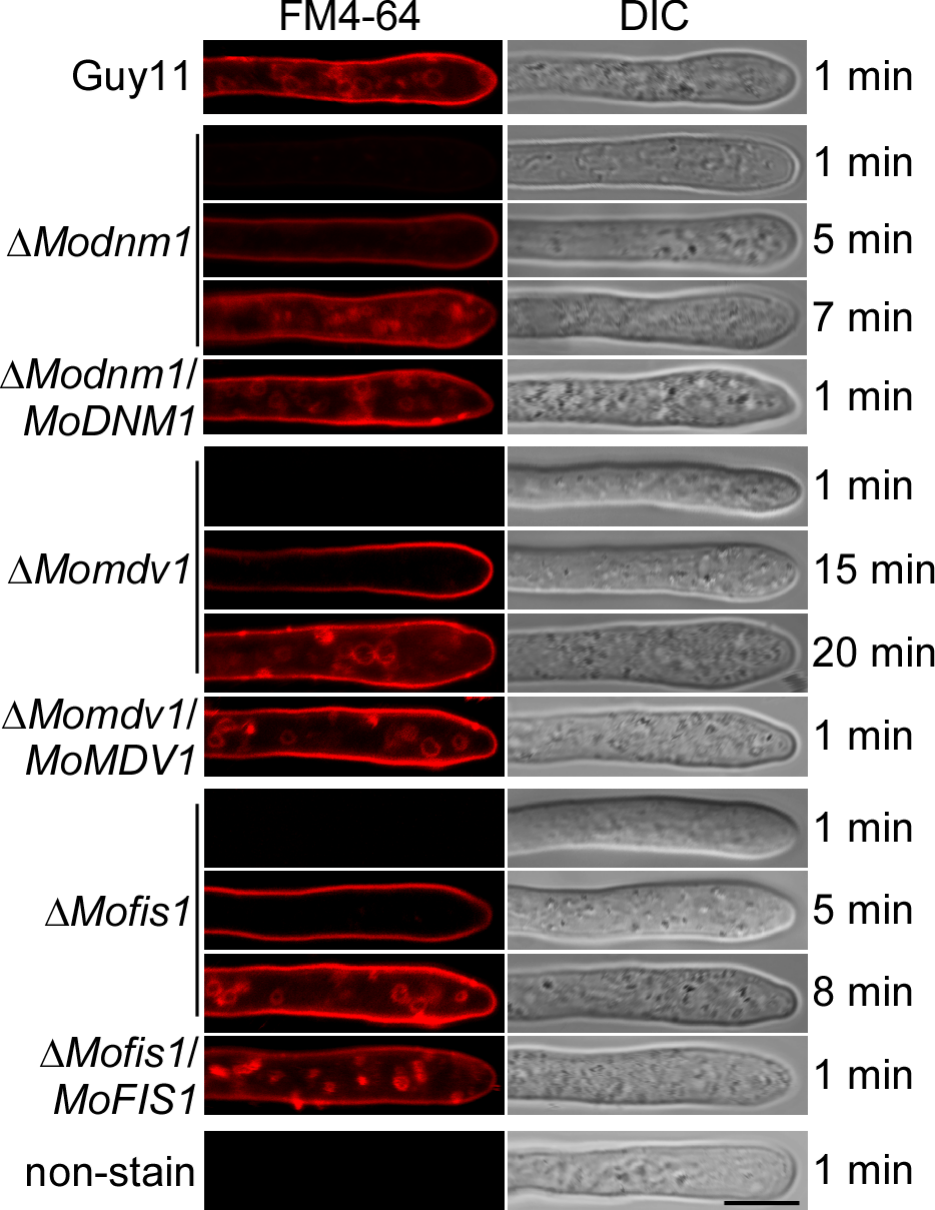
MoDnm1, MoMdv1 and MoFis1 are required for normal endocytosis. Hyphae of the indicated strains were cultured in liquid CM for 40 h, then stained with FM4-64 and examined under confocal fluorescence microscope (Zeiss LSM710, 63x oil). Bar = 5 μm.

Endocytosis is the process through which cells internalize portions of their plasma membrane along with extracellular material and it is fundamentally important in cell function. The dynamin-linked GTPase function has a critical role in membrane remodeling and endocytic membrane fission events [3]. To investigate whether the loss of MoDnm1 results in defects in endocytosis and intracellular transport, we observed endocytosis by staining the hyphae with FM4-64. We found that FM4-64 was internalized within 1 min after exposure in wild type, but a delay of approximate 7 min in the Δ*Modnm1* mutant (Fig 5). Like MoDnm1, MoFis1 and MoMdv1 were also found to be required for endocytosis, as agreed by the similar delay in FM4-64 internalization. Following exposure, the dye was not detected until up to 20 min in the Δ*Momdv1* mutant and 8 min in the Δ*Mofis1* mutant (Fig 5).

### MoDnm1 is localized to peroxisomes and mitochondria

**Fig 6:**
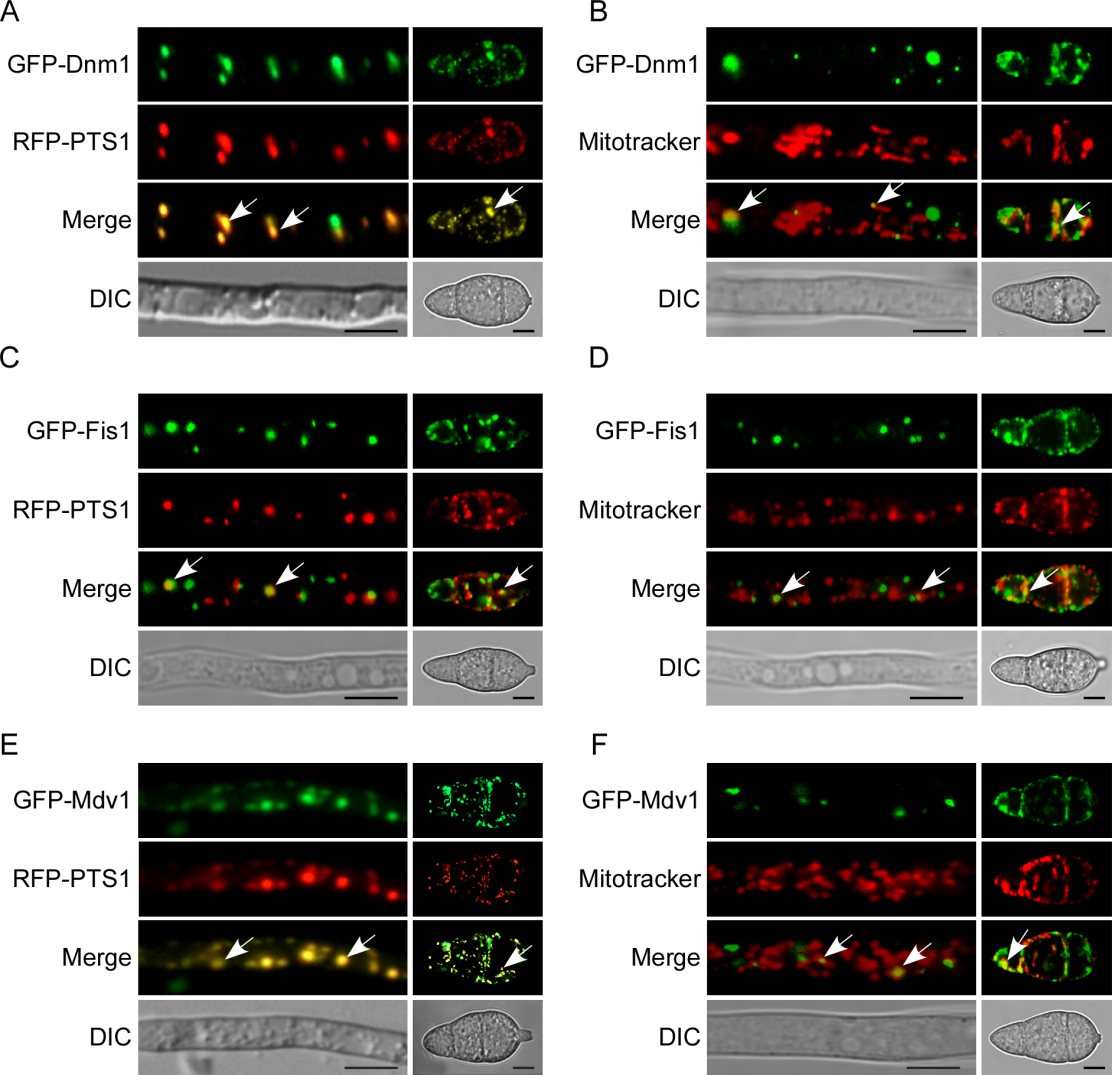
MoDnm1, MoFis1, and MoMdv1 is localized to both peroxisomes and mitochondria. (A, C and E) Confocal fluorescence microscope (Zeiss LSM710, 63x oil) observation showing that MoDnm1, MoFis1, and MoMdv1 co-localized with peroxisome marker RFP–PTS1 in both hyphae and conidia. Arrows denote overlapped GFP and RFP Fluorescence signal. Bar = 5 μm. (B, D and F) Confocal fluorescence microscope (Zeiss LSM710, 63x oil) observation showing that MoDnm1, MoFis1 and MoMdv1 co-localized with the mitochondrial marker MitoTracker in both hyphae and conidia. Arrows denote co-localized GFP and RFP Fluorescence signal. Bar = 5 μm.

To further characterize MoDnm1 functions, we examined the subcellular localization of MoDnm1. MoDnm1 was fused with a GFP tag at the N-terminus (GFP-Dnm1) and expressed in the Δ*Modnm1* mutant. Punctate green fluorescence was observed in both vegetative hyphae and conidia. To test if GFP-Dnm1 is located in organelles, an RFP-PTS1 (PTS1 encodes peroxisomal targeting signal 1) was introduced into the same strain. Red and green fluorescence was detected and overlapped (Fig 6A), suggesting that MoDnm1 is localized to the peroxisomes. In addition, hyphae and conidia of the Δ*Modnm1* mutant transformed with GFP-Dnm1 were incubated with a far red-fluorescent dye (MitoTracker, abs/em ~644/665 nm) that stains mitochondria in live cells. As shown in Fig 6B, GFP fluorescence was observed as distinct spots partially co-localized with the mitochondrial structures, in line with Dnm1p localizations reported in other species [26]. These results indicate that MoDnm1 is localized to both peroxisomes and mitochondria.

Since the mammalian Dlp1 and *S*. *cerevisiae* Dnm1 proteins mediate peroxisomal and mitochondrial fission in complex with Fis1 and Mdv1 proteins [24, 26, 29–31, 53], we also tested the localizations of MoFis1 and MoMdv1 using the same method. Fluorescence signal of GFP and RFP was detected and overlapped, indicating that both MoFis1 and MoMdv1 are located to the peroxisomes. Moreover, studies of mitochondria localization of these two proteins revealed that both MoFis1 and MoMdv1 are partially co-localized with mitochondria (Fig 6C–6F), consistent with an earlier report [58]. Taken together, the observation of colocalization supports that MoDnm1, MoFis1, and MoMdv1 function as a complex.

### MoDnm1 is important for peroxisomal and mitochondrial fission

**Fig 7:**
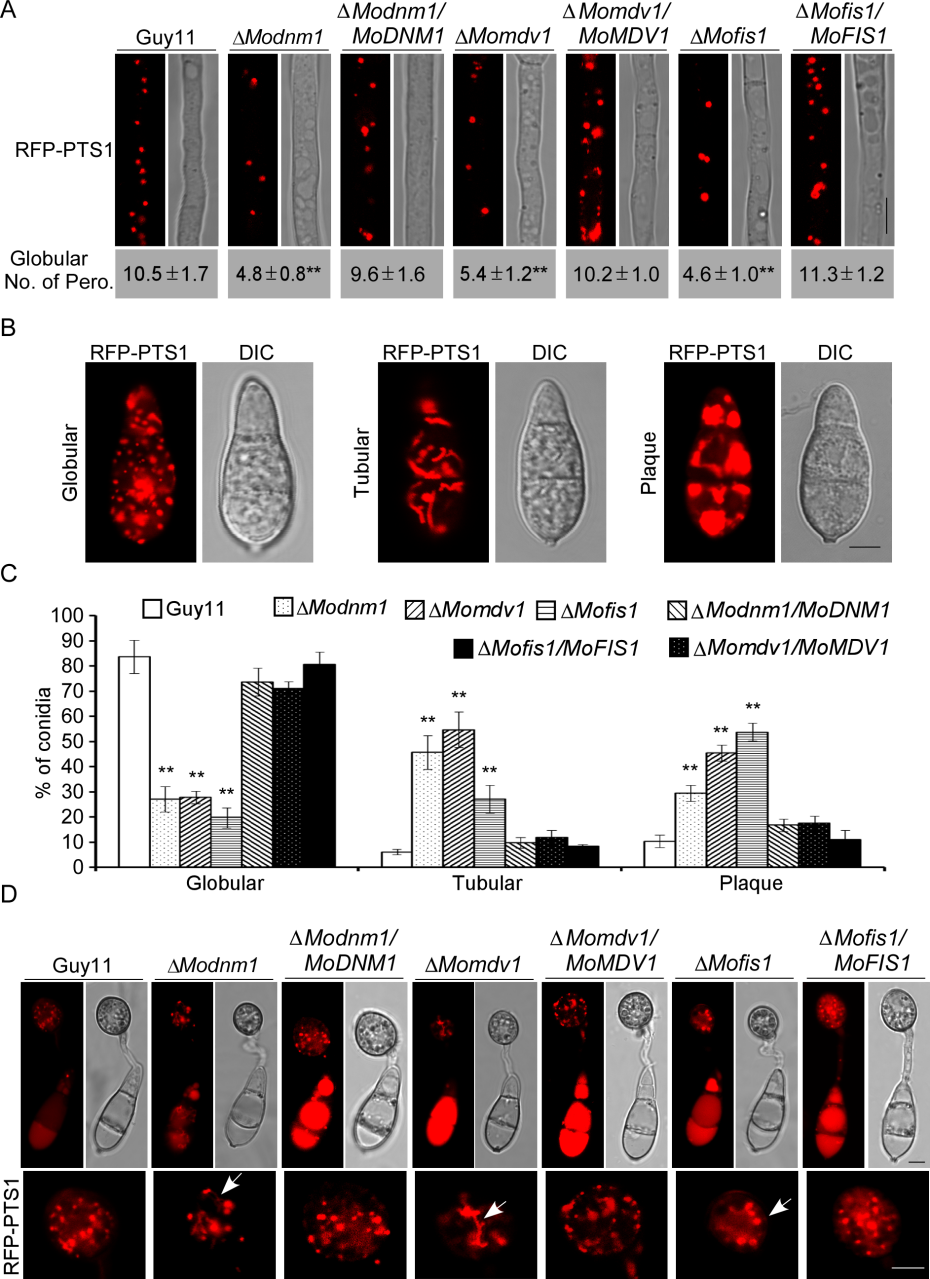
MoDnm1, MoMdv1, and MoFis1 are important for peroxisomal fission. (A) Observation and statistical analysis of the peroxisomes in hyphae and conidia of the transformants. Asterisks denote statistical significances (Duncan's new multiple range test, *p*<0.01). Bar = 5 μm. (B and C) Observation and statistical analysis of the peroxisomes in conidia of different indicated strains. Asterisks denote statistical significances (Duncan's new multiple range test, *p*<0.01). Bar = 5 μm. (D) Observation of the peroxisomes in appressoria of indicated strains. Arrows denote tubular morphology peroxisomes. Bar = 5 μm. Observations were made with a confocal fluorescence microscope (Leica TCS SP8, 100x oil).

**Fig 8:**
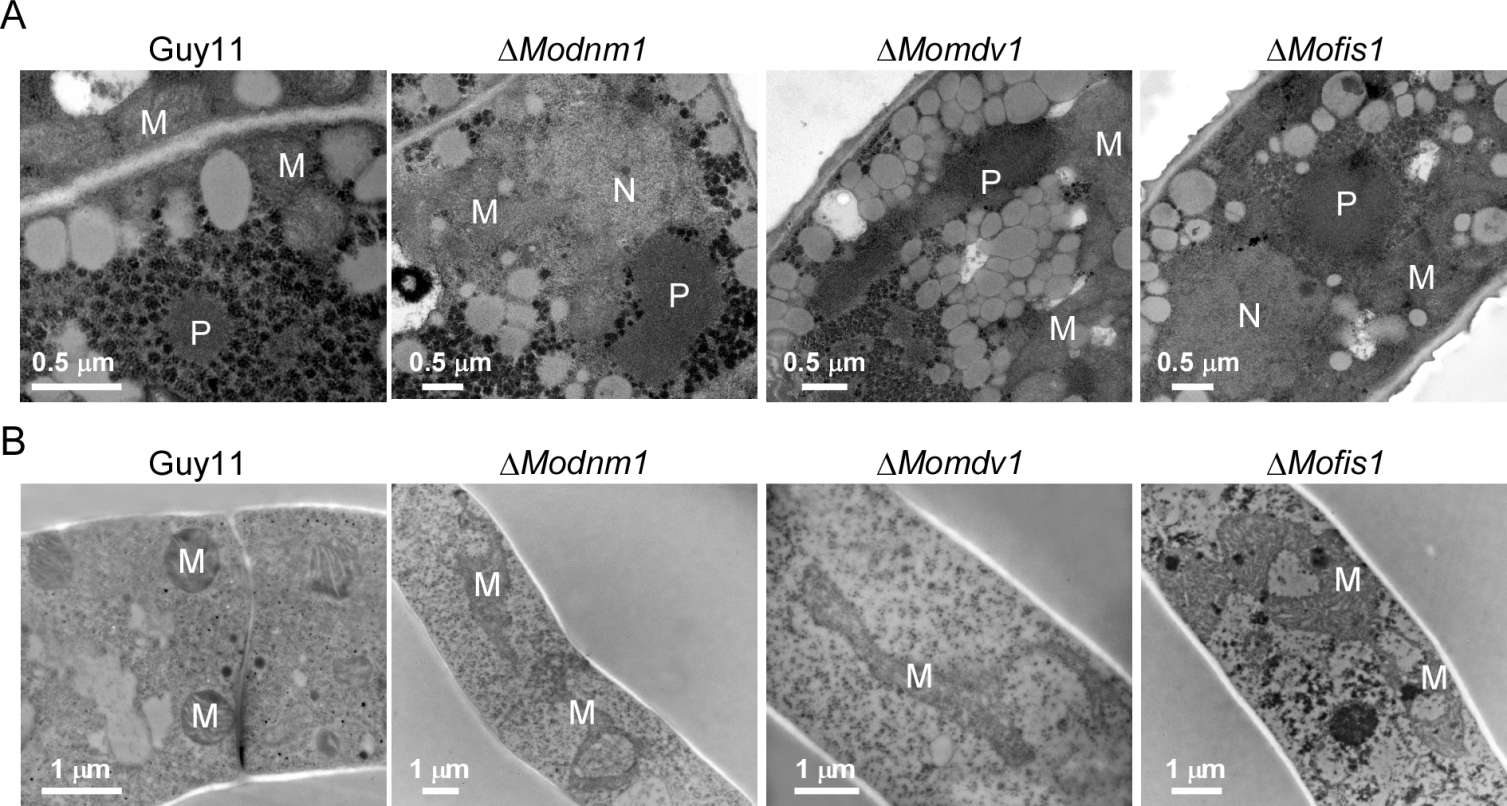
MoDnm1, MoMdv1, and MoFis1 are important for peroxisomal and mitochondrial morphology. (A) Transmission electron microscopy (Hitachi H-7650) observation of peroxisomes in conidia of the indicated strains. Bar = 0.5 μm. P, peroxisome; M, mitochondria; N, nucleus. (B) Transmission electron microscopy (Hitachi H-7650) observation of mitochondria in hyphae of the indicated strains. Bar = 1 μm. M, mitochondria.

To examine functions of MoDnm1, MoMdv1, and MoFis1 in peroxisomal fission, we expressed RFP-PTS1 in the wild type, Δ*Modnm1*, Δ*Mofis1*, Δ*Momdv1* mutants, and the complemented strains Microscopic observations showed the presence of independent punctate pattern of RFP-PTS1 in hyphae of all strains, but the numbers of peroxisomes were markedly reduced in the Δ*Modnm1*, Δ*Mofis1* and Δ*Momdv1* mutants (Fig 7A). In addition, all three deletion mutants showed a higher percentage of peroxisomes with plaque and tubular morphology in conidia in comparison to those of the wild type and the complemented strains (Fig 7B and 7C). Furthermore, we observed the morphology of peroxisomes in appressoria and found more tubular peroxisomes and less globular peroxisomes (Fig 7D). Transmission electron microscopy (TEM) was finally used to compare the impaired fission on peroxisome organization in the mutants. Peroxisomes in the Δ*Modnm1*, Δ*Mofis1* and Δ*Momdv1* mutants appeared dramatically enlarged compared to the wild type (Fig 8A). The decreased peroxisome numbers and altered peroxisomal morphology suggest that MoDnm1, MoMdv1 and MoFis1 are key regulators of peroxisomal proliferation.

**Fig 9:**
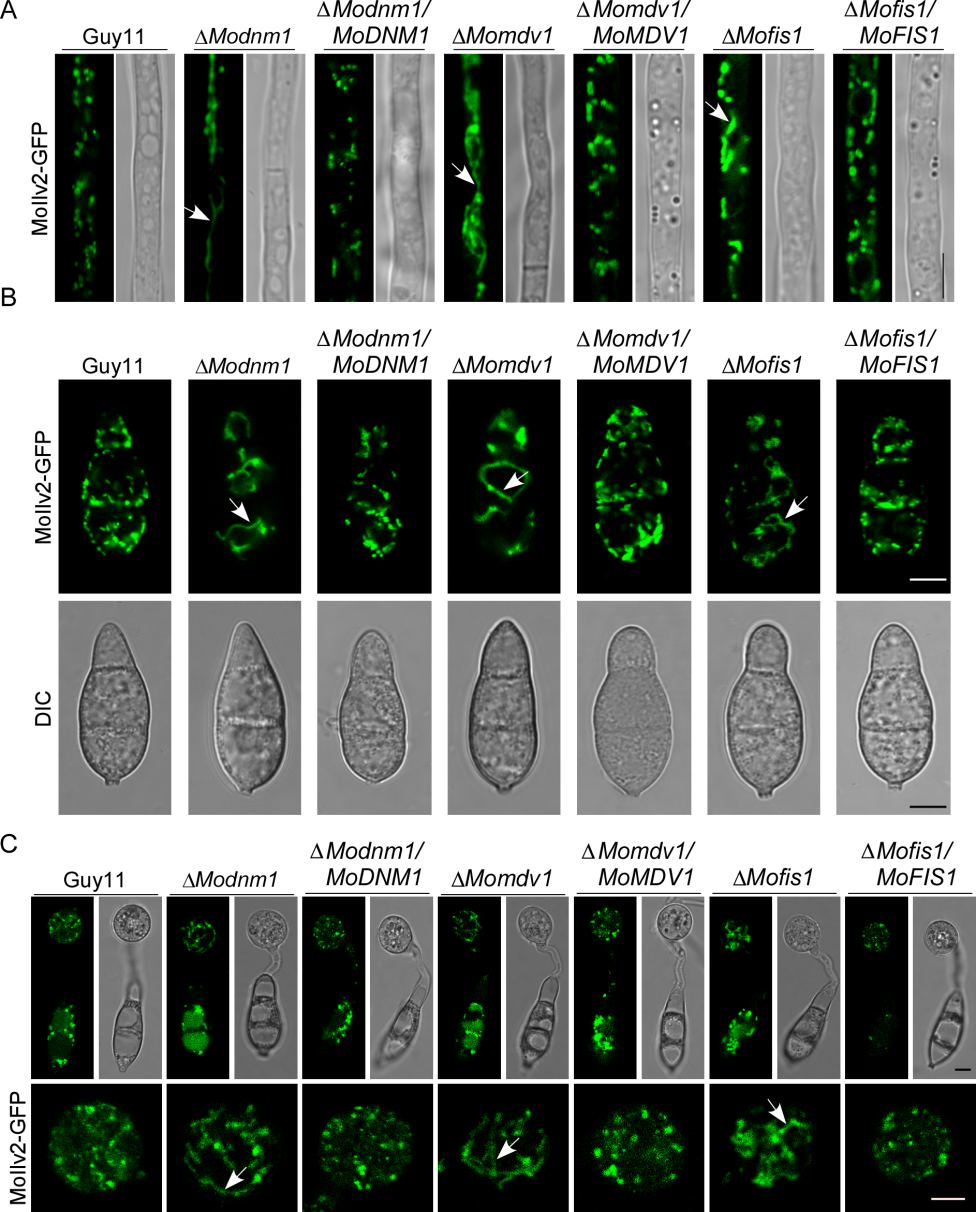
MoDnm1, MoMdv1, and MoFis1 are all important for mitochondrial fission. (A, B and C) Observation of the mitochondria in hyphae, conidia, and appressoria of the indicated strains using a Leica confocal fluorescence microscope (Leica TCS SP8, 100x oil). Arrows denote tubular morphology peroxisomes. Bar = 5 μm.

In addition, we expressed the mitochondria localization fusion protein MoIlv2-GFP in all strains to determine mitochondrial fission conditions. Green fluorescence that indicates mitochondria was observed as an elongated tubule in hyphae, conidia and appressoria of the deletion mutants, but as punctate patterns in different stages of the wild type and the complemented strains (Fig 9A–9C). Electron microscopic comparison revealed that mitochondria in the Δ*Modnm1*, Δ*Mofis1*, and Δ*Momdv1* mutants all appeared dramatically enlarged and elongated (Fig 8B). Taken together, these results suggest that MoDnm1 mediates peroxisomal and mitochondrial fission in complex with MoMdv1 and MoFis1.

### Impaired peroxisomal and mitochondrial fission result in pathogenicity reduction

**Fig 10:**
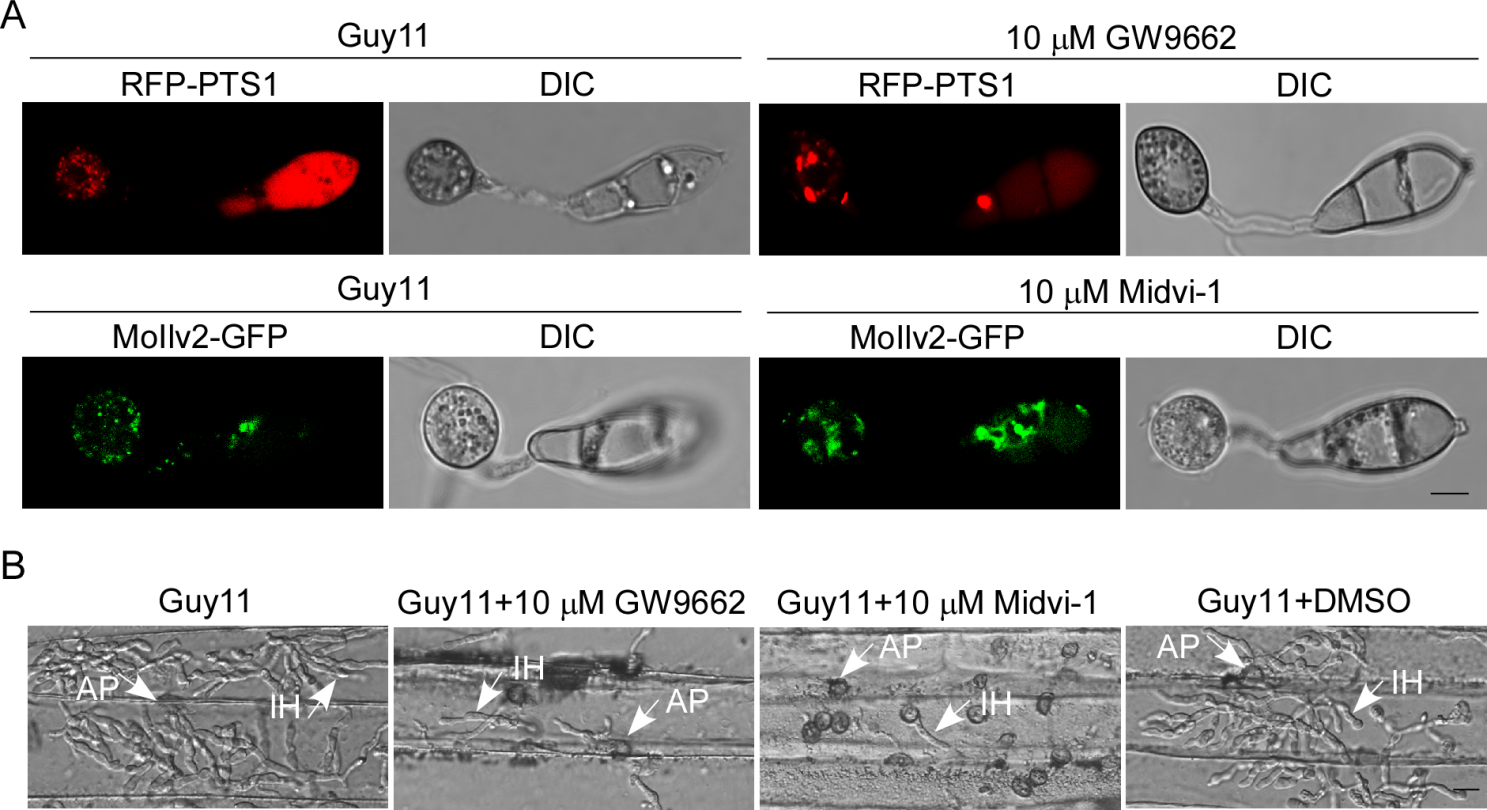
Peroxisomal and mitochondrial fission are required for infection. (A) Observation of the peroxisomes and mitochondria in appressoria of the wild type strain after incubation with inhibitors of peroxisomal fission (10 μM GW9662) and mitochondrial fission (10 μM Midvi-1) using confocal fluorescence microscope (Leica TCS SP8, 100x oil). Bar = 5 μm. (B) Observation for infectious growth on barley leaves at 24 hpi using fluorescent microscopy (Zeiss Axio Observer A1 20x). Guy11+DMSO denote a negative control. Arrows denote appressorium (AP) and invasive hyphae (IH). Bar = 10 μm.

To reveal the underlying role of dynamins and the fission complex in pathogenicity, we further studied the role of peroxisomal and mitochondrial fission in appressorial functions. We treated the appressoria of the wild type strain with the peroxisomal fission inhibitor, GW9662 [59] and mitochondrial fission inhibitor Midvi-1 [60], respectively. Fewer and enlarged peroxisomes were observed in appressoria after incubation with GW9662 for 24 h, and fewer mitochondrial punctate patterns were observed after incubation with Midvi-1 for 24 h (Fig 10A). In addition, the pathogenicity was significantly reduced after incubation with either GW9662 or Midvi-1 for 24 h (Fig 10B). All these results are consistent with that both peroxisomal and mitochondrial fission are required for infection.

To explore whether the attenuated virulence in Δ*Modnm1*, Δ*Momdv1*, and Δ*Mofis1* mutants is due to defects in peroxisomal and mitochondrial fission, appressoria from the mutants and wild type strain were treated with oleate and glycerol to induce peroxisomal and mitochondrial fission, respectively [32, 61–63]. Globular peroxisomes and mitochondria were markedly increased in deletion mutants as well as in the wild type after treated with 0.1% V/V oleate or 1% V/V glycerol for 10 h, respectively (S5A–S5D Fig). In addition, the post treatment conidia of deletion mutants and the wild type were drop-inoculated to barley. The wild type treated with both inducers showed at least a 30% increase of type 4 infectious hyphae at 20 hpi. The Δ*Modnm1*, Δ*Momdv1*, and Δ*Mofis1* mutants treated with oleate showed about 19, 37 and 45% increase of type 4 infectious hyphae at 26 hpi, and treated with glycerol showed 16, 27 and 42% increase of type 4 infectious hyphae, respectively (S5E and S5F Fig). The increased virulence by inducing peroxisomal and mitochondrial fission suggests that the loss of appressorial function in Δ*Modnm1*, Δ*Momdv1*, and Δ*Mofis1* mutants is partially due to impaired peroxisomal and mitochondrial fission.

**Fig 11:**
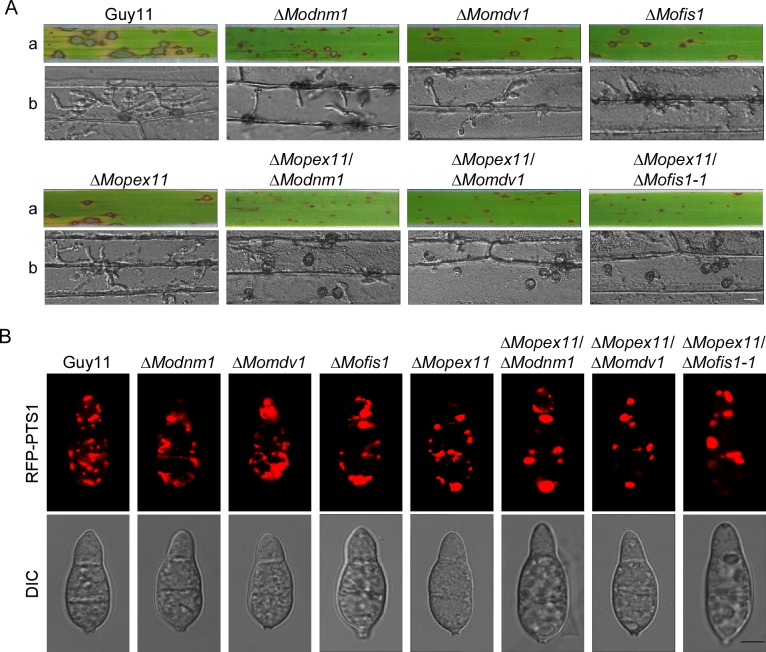
MoPex11A, MoDnm1, MoMdv1, and MoFis1 all have functions on peroxisomal fission. (A) a, Disease symptoms on the rice seedlings which were sprayed with conidial suspensions at 7 dpi. b, Disease symptoms on the detached barley which were drop-inoculated with conidial suspensions and examined at 24 hpi. Bar = 10 μm. (B) Observation of peroxisomal morphology in the indicated strains using confocal fluorescence microscope (Leica TCS SP8, 100x oil). Bar = 5 μm.

To validate that the peroxisomal proliferation is important for infection, we deleted the *PEX11A* gene [14] in the Δ*Modnm1*, Δ*Mofis1*, and Δ*Momdv1* mutants and characterized these mutants (S4A–S4C Fig). They all showed similar growth rates to the Δ*Modnm1*, Δ*Mofis1*, and Δ*Momdv1* mutants, respectively (S4D Fig). The double mutants showed a significant reduction in virulence compared to the single mutant lines in the assays using rice and detached barley leaves for close examination (Fig 11A). These results demonstrate that all three double deletion mutants have more severe defects in virulence than that in the single deletion mutants. In addition, elongated conidia were observed in the Δ*Mopex11A/*Δ*Mofis1* and Δ*Mopex11A/*Δ*Modnm1* mutants (S4E Fig). Furthermore, to observe whether peroxisomal proliferation was inhibited in these double deletion mutants, RFP-PTS1 was expressed in these strains. Peroxisomes showed plaque-like morphology in all three double deletion mutants similar to that in Δ*Mopex11A* mutant (Fig 11B). These results indicate that MoPex11A, MoDnm1, MoMdv1, and MoFis1 all have functions in peroxisomal proliferation but in different ways.

Mitochondria are major compartments for the TCA cycle to supply energy while peroxisomes are required for fatty acid degradation and the glyoxylate cycle [64]. To investigate whether abnormal mitochondrial fission would result in lack of energy for penetration and extending, exogenous ATP was added in conidia suspensions of the deletion mutants before infection. However, this did not rescue the virulence of the Δ*Modnm1*, Δ*Momdv1* or Δ*Mofis1* mutants (S6A Fig). HPLC assays also showed that these three deletion mutants contain similar concentration of ATP when compared with the Guy11 strains in mycelia (S6B Fig). Therefore, these results suggest that the Dnm1-mediated alteration of mitochondria morphology might have no effect on ATP production.

### MoDnm1, MoMdv1, and MoFis1 are involved in pexophagy, mitophagy and autophagy

**Fig 12:**
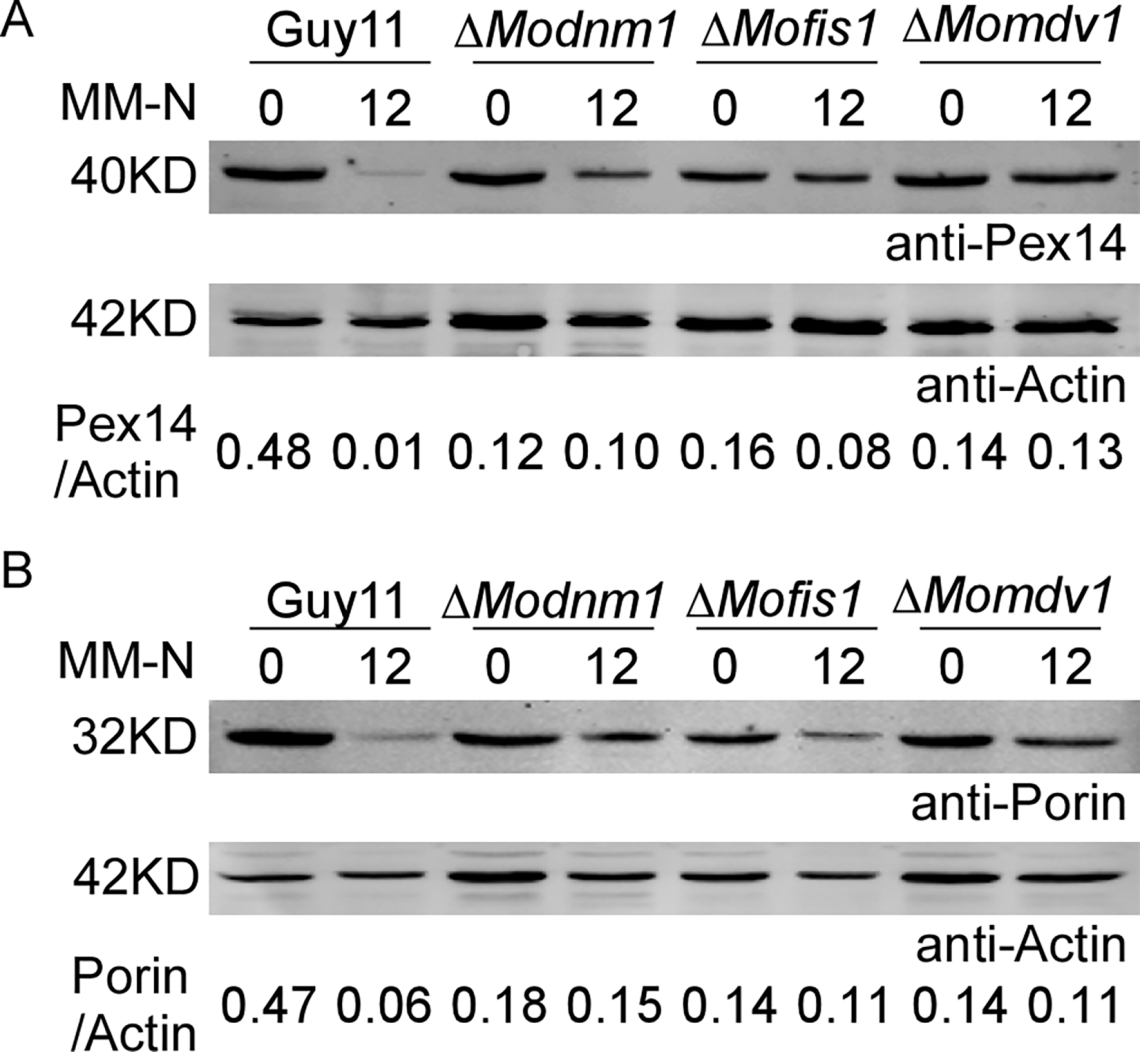
MoDnm1, MoMdv1 and MoFis1 are involved in mitophagy and pexophagy. (A) The indicated strains were cultured in liquid CM for 40 h, then shifted to BM-O for 20 h and starved in MM-N for 12 h. Cells were collected and the cell lysates were subjected to immunoblot analysis with the anti-Pex14 antibody. The amount of stable Pex14 was monitored by relative ratios of Pex14 and Actin (underneath the blot). Densitometric analysis was performed by Image-pro plus (Media Cybernetics Inc., Shanghai, China). (B) The indicated strains were cultured in liquid CM for 40 h, then shifted to BM-G for 30 h and starved in MM-N for 12 h. Mycelia were harvested and the cell lysates were subjected to immunoblot analysis with the anti-Porin antibody. The amount of stable porin was represented by relative ratios of porin and actin (underneath the blot). Densitometric analysis was performed by Image-pro plus (Media Cybernetics Inc., Shanghai, China).

Pexophagy and mitophagy are cellular process to selectively remove peroxisomes and mitochondria through autophagy [42, 65], and peroxisomal and mitochondrial fission are required for pexophagy and mitophagy [49, 66]. Since peroxisomal and mitochondrial fission were severely inhibited in Δ*Modnm1*, Δ*Mofis1*, and Δ*Momdv1* mutants, we determined whether these three proteins are required for selective autophagy, mitophagy and pexophagy. Pexophagy was monitored based on the amount of stable Pex14 protein, a peroxisomal integral membrane protein by immunoblot analysis. When pexophagy is induced, peroxisomes, along with Pex14, are delivered into the vacuole for degradation. As a result, fewer stable Pex14 was detectable in the Δ*Modnm1*, Δ*Mofis1*, and Δ*Momdv1* mutants than that in the wild type (Fig 12A). Similarly, mitophagy was monitored by assessing the total amount of stable porin, a mitochondrial marker protein by immunoblot analysis [61], and few stable porin was detected in the Δ*Modnm1*, Δ*Mofis1*, and Δ*Momdv1* mutants than that in the wild type (Fig 12B). Overall, these results suggest that MoDnm1, MoFis1, and MoMdv1 are important for mitophagy and pexophagy.

**Fig 13:**
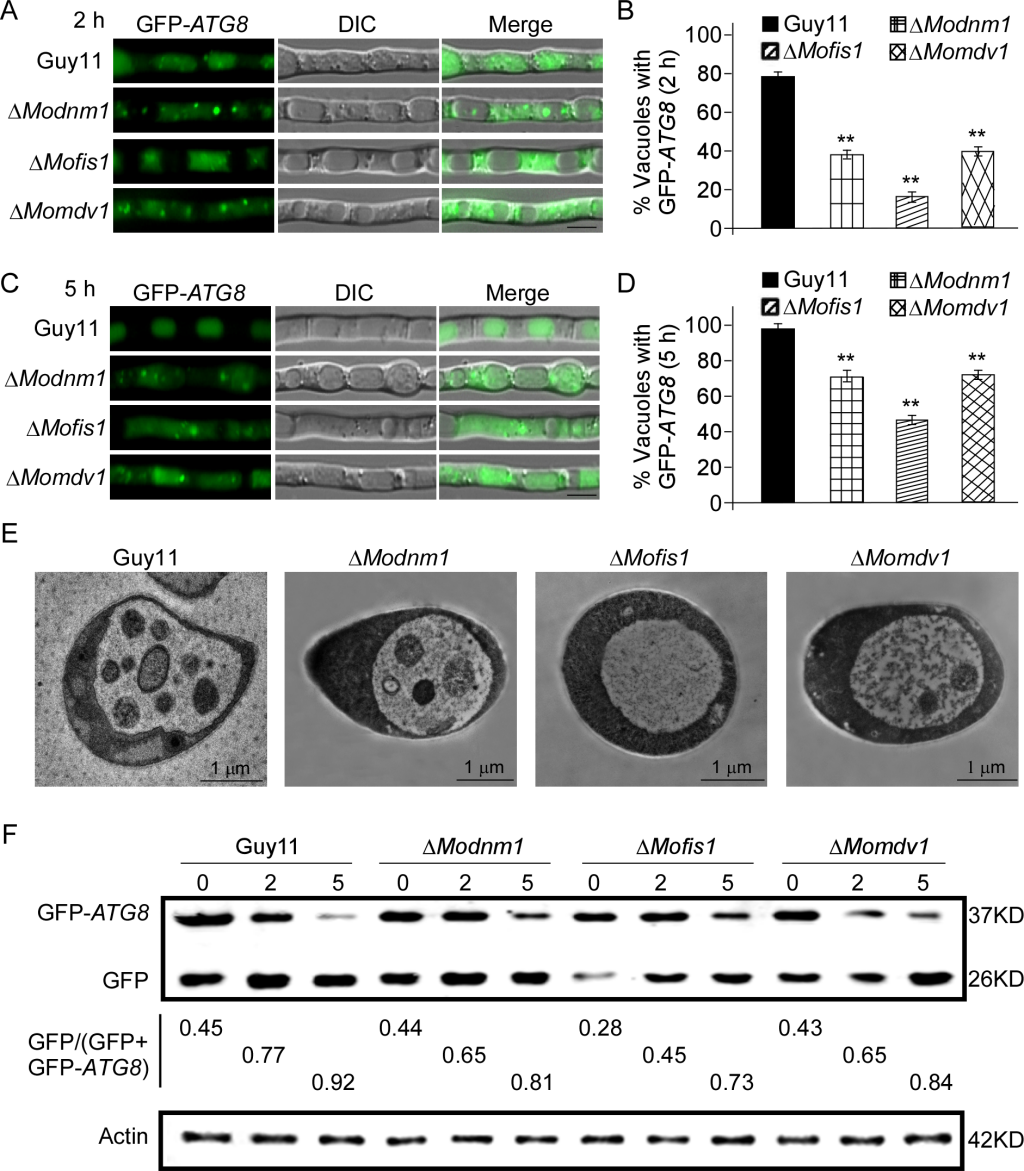
MoDnm1, MoMdv1 and MoFis1 are involved in autophagy. (A and C) The wild type, Δ*Modnm1*, Δ*Mofis1*, and Δ*Momdv1* strains transformed with GFP-Atg8 were cultured in MM-N (nitrogen starvation minimal medium) for 2 h or 5 h, and the autophagy activity was observed by Axio Observer A1 Zeiss inverted microscope. (B and D) Autophagy activity was assessed by means of translocation of GFP-Atg8 into vacuoles (n = 100). Bars with asterisks represent significant differences (Duncan's new multiple range method *p*<0.01). Bar = 5μm. (E) Transmission electron microscopy (Hitachi H-7650) observation of hyphae cultured in nitrogen starvation MM–N medium for 5 h. Bar = 1 μm. (F) Immunoblotting was performed with anti-GFP and anti-*β*-Actin antibodies. The extent of autophagy was estimated by calculating the amount of free GFP compared with the total amount of intact GFP-Atg8 and free GFP (the numbers underneath the blot). Densitometric analysis was performed by Image-pro plus (Media Cybernetics Inc., Shanghai, China).

We next determined whether MoDnm1, MoMdv1, and MoFis1 are involved in nonspecific autophagy. GFP-Atg8 was expressed in the wild type, Δ*Modnm1*, Δ*Mofis1*, and Δ*Momdv1* strains to monitor nonspecific autophagy. When autophagy is induced, autophagic bodies, along with GFP-Atg8, are delivered into the vacuole for degradation. In the Δ*Modnm1*, Δ*Mofis1*, and Δ*Momdv1* mutants, a vacuolar GFP signal was detected after starvation in MM-N for 2 or 5 h, with a reduced number of vacuole (2 h: 38, 17 and 42%, 5 h: 71, 49 and 74%, respectively) containing autophagic bodies, compared to that of the wild type strain (2 h: 80%, 5 h: 98%) (Fig 13A–13D). To further confirm this, autophagic bodies were observed using Transmission electron microscopy after culturing in MM-N with 2 mM PMSF (Phenylmethanesulfonyl fluoride) for 4 h [63]. All these three mutants accumulated less autophagic bodies in the lumen of vacuoles within hyphae than that of the wild type (Fig 13E). Autophagy was monitored based on the amount of free GFP by immunoblot. In the wild type, a considerable amount of free GFP was detected after 5 h nitrogen starvation. The amount of free GFP was reduced in Δ*Modnm1* and Δ*Momdv1* mutants, and dramatically reduced in Δ*Mofis1* mutant. The extent of autophagy was estimated by calculating the amount of free GFP compared with the total amount of intact GFP-Atg8 and free GFP. Consistent with the microscopic observations, the Δ*Modnm1*, Δ*Mofis1*, and Δ*Momdv1* mutants showed the delayed nonspecific autophagy and the higher stabilized levels of GFP-Atg8 at the same time points compared with the wild type (Fig 13F). Thus, we conclude that MoDnm1, MoMdv1, and MoFis1 are all involved in nonspecific autophagy of *M*. *oryzae*.

### MoDnm1 and MoFis1 domain-specific functions

**Fig 14:**
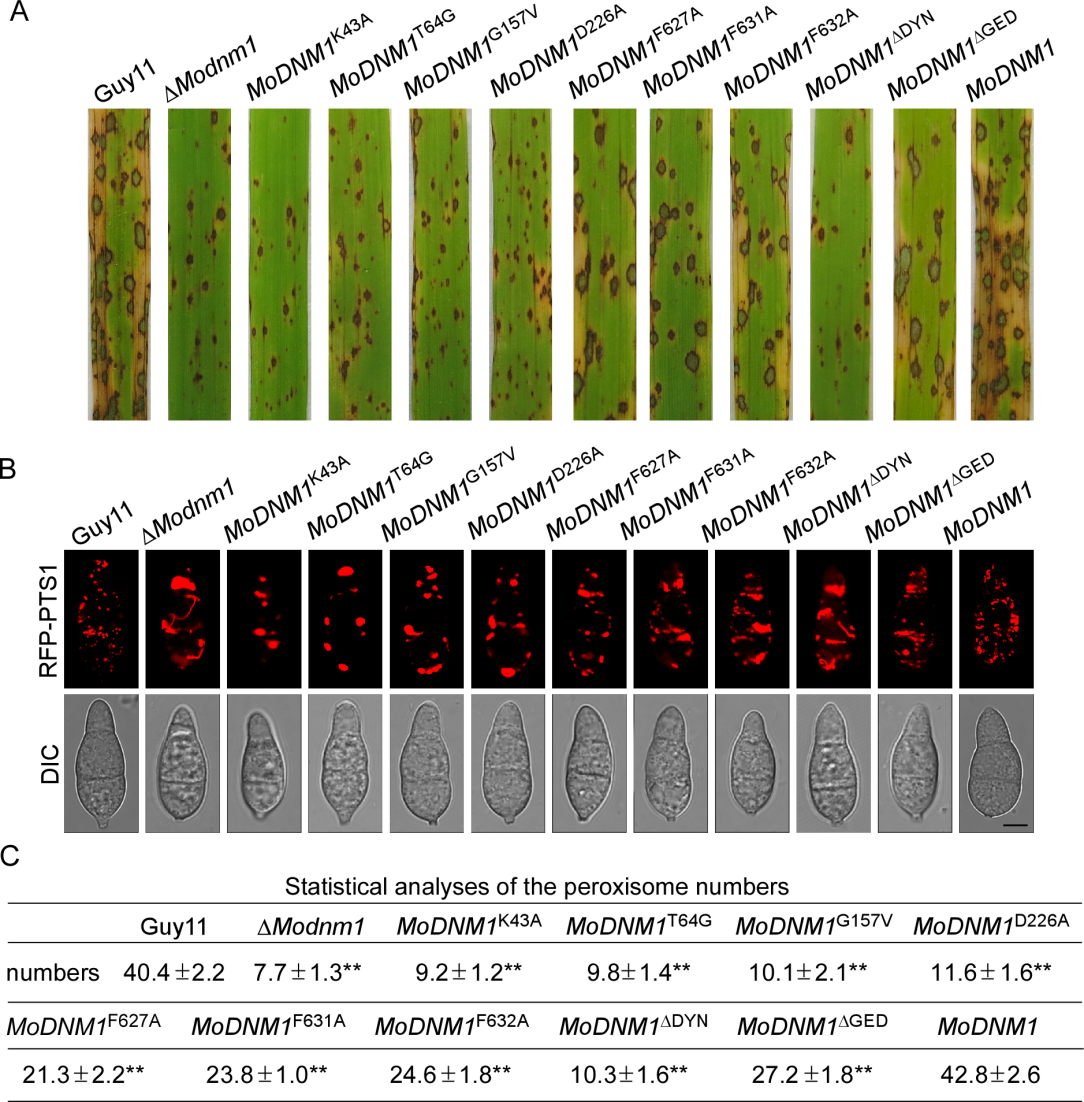
The InsB motif, DYNc and GED domains of MoDnm1 are important for Pathogenicity and peroxisomal fission. (A) Disease symptoms on rice seedlings sprayed with conidial suspensions at 7 dpi. (B) Observation of peroxisomal morphology in the indicated strains by confocal fluorescence microscope (Leica TCS SP8, 100x oil). Bar = 5 μm. (C) Statistical analysis of peroxisome numbers of different strains. The number of peroxisomes was counted by Image-pro plus software (Media Cybernetics Inc., Shanghai, China). ±SD was calculated from three repeated experiments and asterisks indicate statistically significant differences (Duncan's new multiple range test, *p*<0.01).

Dynamins are large GTPases with conserved GTPase domain, middle domain and the GED domain. The GTPase domain contains the GTP-binding motifs (G1–G4) that are required for guanine-nucleotide binding and hydrolysis. Only one GTP molecule can be bound to each GTPase domain, but the sequences that contribute to the interactions are spread over the domain [1]. The key residues of MoDnm1 are shown in S7A Fig. The G1 motif (in the so-called P-loop) coordinates the phosphates, whereas the threonine in the G2 motif is involved in catalysis. The glycine in the G3 motif forms a hydrogen bond with the γ-phosphate of GTP and the G4 motif is involved in base and ribose coordination [1]. In place of the PH domain, an InsB motif was identified in *S*. *cerevisiae* Dnm1. The amino acid sequence of this motif is strictly conserved among fungi and amino acid substitutions in this InsB helix inhibit the recruitment of Dnm1 to mitochondria and block fission [67]. The key residues of InsB are shown in S7B and S7C Fig. In this study, we generated seven point mutation mutants *MoDNM1* ^K43A^, *MoDNM1* ^T64G^, *MoDNM1* ^G157V^, *MoDNM1* ^D226A^, *MoDNM1* ^F627A^, *MoDNM1* ^F631A^, *MoDNM1* ^F632A^, and two main function domain deletion mutants *MoDNM1* ^ΔDYN^ and *MoDNM1* ^ΔGED^ (S7D Fig). Compared with the wild type and the complement strains (*MoDNM1*), the growth rates of all these mutants were significantly reduced (S8A Fig). On the other hand, the InsB motif point mutation mutants and GED deletion mutants showed restored virulence similar to that of Δ*Modnm1* mutant and the complement strain (*MoDNM1*) (Fig 14A). Similar to Δ*Modnm1* conidia suspensions, very few and small lesions developed at 7 dpi with conidia suspensions of G1, G2, G3, G4 motifs point mutation mutants and the DYNc domain deletion mutants (Fig 14A). Quantification of the *M*. *oryzae* biomass in rice using a ‘relative fungal growth’ assay by qRT-PCR showed a similar result. The InsB motif point mutation mutants and GED deletion mutants showed a virulence complementation up to 70% of the wild type (S8B Fig), while other mutants showed similar virulence as the Δ*Modnm1* mutant. These results indicate that G1 to G4 box have critical role in *M*. *oryzae* growth and pathogenicity, and the InsB motif has partial functions on growth and pathogenicity. In addition, we observed peroxisomal morphology in all these point mutation mutants. In contrast to the abundant globular peroxisomes in the wild type, the morphology of peroxisomes in point mutation mutants changed to plaque or tubular (Fig 14B). The number of peroxisomes was significantly reduced in all point mutation mutants, and G1, G2, G3, G4 motifs point mutation mutants and GED deletion mutants showed more severe inhibition of peroxisome fission. These results suggest that G1 to G4 boxes have critical role on peroxisomal fission and the InsB motif has partial functions on peroxisomal fission.

As the short C-terminal tail of hFis1 is both necessary and sufficient for its targeting to peroxisomes, whereas the N-terminal region is required for organelle fission [31], we examined the role of MoFis1 N-terminal region in fission in *M*. *oryzae*. We expressed three N-terminally truncated MoFis1 constructs MoFis1^Δ1–29^ (Δ1–29), MoFis1^Δ1–57^ (Δ1–57), and MoFis1^Δ1–88^ (Δ1–88) in Δ*Mofis1* with RFP-PTS1 cells. In contrast to the wild type, aggregation of peroxisomes was observed with the RFP-tagged constructs in these three strains (S9C Fig). Furthermore, the N-terminal region of MoFis1 was essential for its growth and pathogenicity, which is different from the C-terminal tail (S9A and S9B Fig). In addition, we examined the influence of C-terminal modifications of MoFis1 on peroxisomal targeting and morphology. Two C-terminal truncated MoFis1 constructs lacking the eight and twenty-nine amino acids, GFP-MoFis1^Δ148–155^ (Δ148–155) and GFP-MoFis1^Δ127–155^ (Δ127–155) were co-expressed with RFP-PTS1, respectively. The truncated protein showed an aggregated distribution with GFP signals gathered into big plaque, but peroxisomal proliferation was normal in these transfected cells (S9D Fig). Furthermore, we found the short C-terminal tail of MoFis1 was dispensable for vegetative growth and pathogenicity (S9A and S9B Fig). These results suggest that the intact C-terminal structure is required for proper peroxisomal distribution, whereas the N-terminal region is required for vegetative growth, peroxisomal fission and pathogenicity.

### MoMdv1 contributes to the peroxisomal localization of MoDnm1 and MoFis1

**Fig 15:**
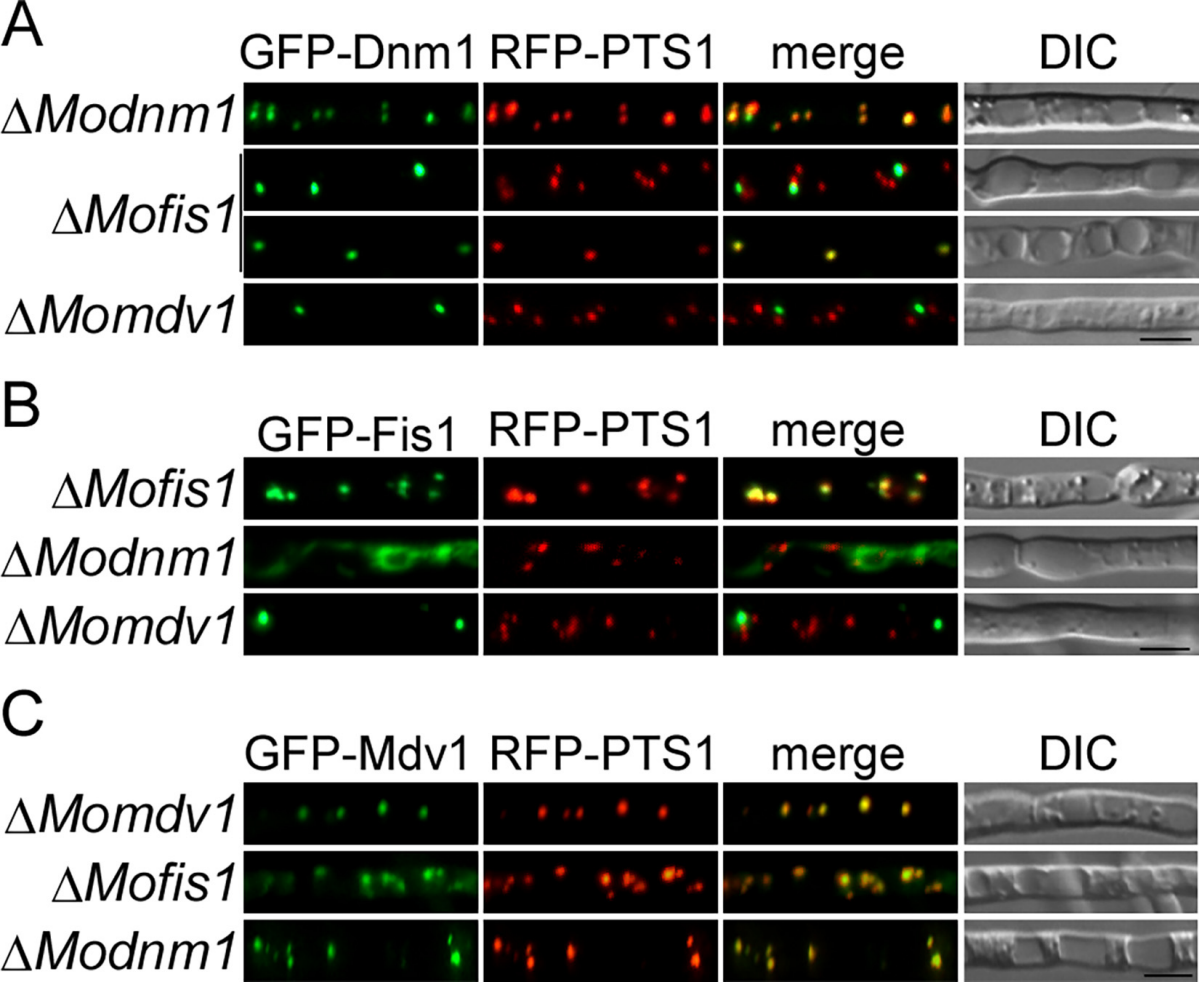
MoMdv1 contributes to the peroxisomal localization of MoDnm1 and MoFis1. (A) GFP-Dnm1 and RFP-PTS1 were co-expressed in Δ*Modnm1* (top), Δ*Mofis1* (middle), and Δ*Momdv1* mutants (bottom). Punctate GFP-Dnm1 localization was observed in hyphae by Axio Observer A1 Zeiss inverted microscope. PTS1, a peroxisome marker protein. Bar = 5 μm. (B) GFP-Fis1 and RFP-PTS1 were co-expressed in Δ*Mofis1* (top), Δ*Modnm1* (middle), and Δ*Momdv1* mutants (bottom). GFP-Fis1 localization was observed in hyphae by Axio Observer A1 Zeiss inverted microscope. PTS1, a peroxisome marker protein. Bar = 5 μm. (C) GFP-Mdv1 and RFP-PTS1 were co-expressed in Δ*Momdv1* (top), Δ*Mofis1* (middle), and Δ*Modnm1* mutants (bottom). GFP-Mdv1 localization was observed in hyphae by Axio Observer A1 Zeiss inverted microscope. PTS1, a peroxisome marker protein. Bar = 5 μm.

As MoMdv1 interacts with both MoDnm1 and MoFis1, we tested whether MoMdv1 mediates the distribution of MoDnm1 and MoFis1 by comparing the localization pattern of MoDnm1 and MoFis1 in the Δ*Momdv1* mutant and the complement strains. Interestingly, in the Δ*Momdv1* mutant, the pattern of GFP-Dnm1 and GFP-Fis1 remained punctate. However, fewer GFP-Dnm1 and GFP-Fis1 punctate structures were observed and these punctate structures failed to localize to peroxisome in comparison to the complement strains (Fig 15A and 15B). In the Δ*Modnm1* and Δ*Mofis1* mutants, the distribution and quantity of Mdv1-containing structures were similar to that in the Δ*Momdv1* complement strains (Fig 15C). We also tested the localization of MoDnm1 in the Δ*Mofis1* mutant and MoFis1 in the Δ*Modnm1* mutant, respectively. In the Δ*Modnm1* mutant, the pattern of GFP-Fis1 was changed to the string shape, but remained localized to peroxisome (Fig 15B). The quantity of GFP-Dnm1 punctate structures was decreased, but remained localized to peroxisome in Δ*Mofis1* mutant (Fig 15A). These observations indicate that the localization of MoDnm1 and MoFis1 to peroxisome requires MoMdv1, and MoMdv1 possesses a Dnm1- or Fis1-independent peroxisome targeting signal.

## Discussion

Membrane transport between compartments in eukaryotic cells requires proteins that mediate the membrane budding and fission events. Classical dynamins and dynamin-related proteins are the essential vesicle-scission molecules [1]. Both are involved in a wide variety of cellular processes including severing endocytic vesicles from the plasma membrane, mitochondrial fission and fusion (Dnm1), vacuolar fission (Vps1), plant cell plate formation, plant cell cytokinesis and membrane fission, and pathogen resistance [1, 6]. In this study, we characterized the functions of main dynamin proteins in *M*. *oryzae*. While previous studies focused more on the functions of dynamins on binding and division of lipid membranes, our studies focused not only on the conserved functions in membrane fission but also on growth and differentiation. Our studies also attempt to establish a novel link between dynamin function on membrane fission and fungal virulence.

In addition to the characterization of the most conserved MoDnm1 protein, we also tackled additional dynamin related proteins and found that they often function in complex. In the budding yeast *S*. *cerevisiae*, four subunits of mitochondrial and peroxisomal fission complex have been identified as Dnm1, Fis1, Mdv1, and Caf4 [22–24]. We also characterized MoFis1 and MoMdv1 and demonstrated that MoMdv1 functions as an adaptor linking MoDnm1 to MoFis1. Further study on the relationship of MoDnm1, MoMdv1, and MoFis1 revealed that MoDnm1 and MoFis1 failed to localize to peroxisome in the Δ*Momdv1* mutant, and the distribution of MoFis1 was also changed dramatically in Δ*Modnm1* mutant. These data are consistent with our hypothesis that MoDnm1 acts as a regulatory molecule through indirect recruiting MoFis1 by an adapter MoMdv1.

In this study, we observed that the Δ*Modnm1*, Δ*Momdv1*, and Δ*Mofis1* mutants exhibited dramatically decreased virulence, which may result from multiple defects of the mutants. First, the vegetative growth rate of the mutant is significantly reduced. This might be a major reason for reduced virulence since the mutant grew significantly slower than the parental strain on different mediums. Second, all these mutants showed a marked decrease in conidiation. This may not be surprising since asexual spores (conidia) are often the major source of primary inoculum and dissemination in phytopathogenic fungi. Third, the Δ*Modnm1*, Δ*Momdv1*, and Δ*Mofis1* mutants have defect in appressorium turgor that leads to the decline of invasion. Following this observation, we further point out that the defect of glycogen degradation was not the reason for lower appressorium turgor in Δ*Modnm1*, Δ*Momdv1*, and Δ*Mofis1* mutants, but rather the fact that peroxisomal and mitochondrial morphology was altered in Δ*Modnm1*, Δ*Momdv1*, and Δ*Mofis1* mutants at different developmental stages. This is consistent with recent studies that showed Pex11A, Pex19 and MoPef1 (Mdv1) are required for peroxisomal proliferation and virulence of *M*. *oryzae* [14, 28, 32].

One of the most important functions of the Modnm1, MoMdv1 and MoFis1 complex is the inhibition of peroxisomal and mitochondrial fission. Lack either one of the complex leads to defects in the formation of small, punctiform peroxisomes and fragmented mitochondria. The studies with Mdivi-1 (mitochondrial division inhibitor), a small molecule, selective inhibitor of DLP1, revealed that inhibition of DLP1 exerts protective effects in heart and cerebral is chemia-reperfusion models and provides neuroprotection in Parkinson models [68]. Our studies with inhibitor Mdivi-1 and inducer oleate demonstrated that the mitochondrial fission in appressorium plays an important role in pathogenicity in *M*. *oryzae*. Similarly, experiments on peroxisomes with peroxisomal fission inhibitor GW9661 and inducer glycerol indicate that peroxisomal fission in appressorium has an important role in pathogenicity. Deletion of *MoPEX11A* leads to attenuated peroxisomal fission and virulence in the rice blast fungus [14]. Here, peroxisomal proliferation is inhibited by deleting *MoPEX11A* in the Δ*Modnm1*, Δ*Momdv1*, or Δ*Mofis1* mutant. The double deletion mutants showed more severe reduction in virulence than single deletion mutants. This observation suggests that the MoDnm1, MoMdv1, and MoFis1 complex mediates peroxisomal fission to affect virulence in this fungus.

In addition to the significantly reduction of peroxisomes and mitochondria, our studies provided evidence that pexophagy, mitophagy and autophagy are delayed in Δ*Modnm1*, Δ*Momdv1*, and Δ*Mofis1* mutants. These results are consistent with the studies in yeast that peroxisomal and mitochondrial fission is important for the degradation [49, 66]. Studies in mammalian cells provide another evidence that normal endocytosis is essential for efficient autophagic flux. The membranes and proteins involved in autophagy initiation and autophagosome precursor formation are internalized by endocytosis [69]. As endocytosis is delayed in Δ*Modnm1*, Δ*Momdv1*, and Δ*Mofis1*, we speculate that the delayed endocytosis could affect the autophagic process. Autophagy is a cellular process involved in various developmental processes, including conidiation and pathogenicity, by assisting carbohydrate metabolism to meet the energy requirements during cellular differentiation [70–72]. Mitochondria are essential for programmed cell death and mitophagy plays an important role in the foot cells during conidiation in *M*. *oryzae* [20, 61]. These results suggest that MoDnm1, in complex with MoMdv1 and MoFis1, is located to peroxisomes and mitochondria to mediate their fission, which underlies processes to regulate conidiation and pathogenicity.

**Fig 16:**
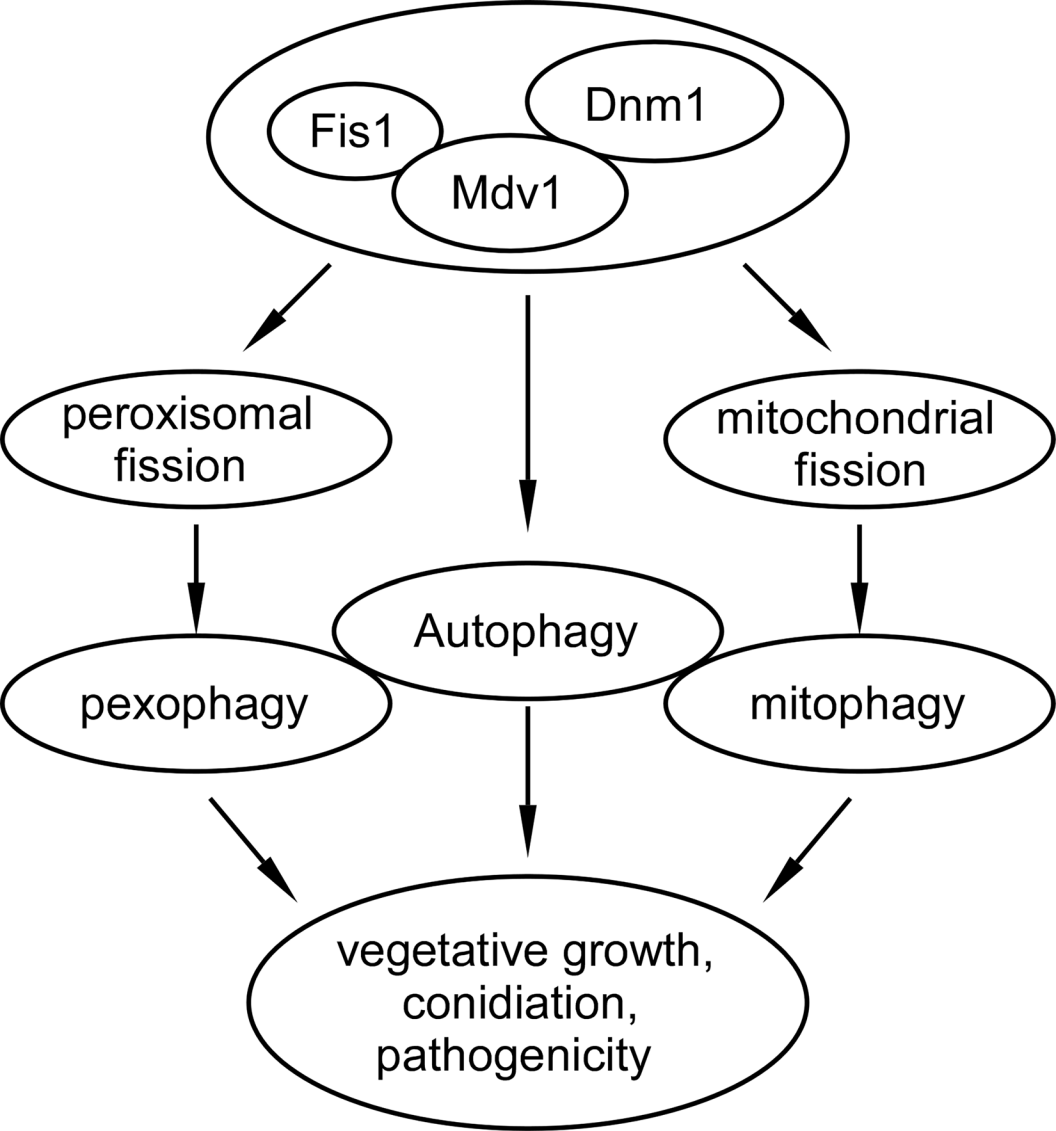
Working model of MoFis1, MoMdv1 and MoDnm1complex in *M*. *oryzae*. The MoDnm1, MoFis1, and MoMdv1 complex mediates peroxisomal and mitochondrial fission to regulate pexophagy and mitophagy required for growth, conidiation and pathogenicity. In parallel, the complex could be involved in the regulation of autophagy that is also important for growth, conidiation, and pathogenicity.

In summary, we presented a working model of MoDnm1 in association with MoFis1 and MoMdv1 in *M*. *oryzae* (Fig 16). MoDnm1, MoFis1, MoMdv1 function as a complex, and MoDnm1 recruits MoFis1 to peroxisome and mitochondria through the adaptor protein MoMdv1. MoDnm1, MoFis1, and MoMdv1 play important roles in asexual development, appressorium function, infectious growth, and pathogenicity. This complex mediates peroxisomal and mitochondrial fission, which may also mediate pexophagy and mitophagy to regulate conidiation and pathogenicity. Ours findings are largely consistent with the functions of Dnm1 and dynamin proteins in *S*. *cerevisiae* and other model organism [49, 66]. However, it is not clear how peroxisomal and mitochondrial fission are required for growth or conidiation in *M*. *oryzae*, thus, further analysis of these proteins will shed lights on the understanding of not only developmental processes but also pathogenesis mechanisms involved in rice blast fungus.

## Materials and Methods

### Strains and culture conditions

The *M*. *oryzae* Guy11 strain was used as wild type (WT) for transformation in this study. For vegetative growth, small agar blocks were cut from the edge of 7-day-old cultures and placed onto fresh media (CM, MM, OM and SDC), followed by incubation in the dark at 28°C. The radial growth was measured after incubation for 7 days [73]. Liquid CM medium was used to prepare mycelia for DNA and protein extraction. For conidiation, strain blocks were maintained on SDC (100 g of straw, 40 g of corn powder, 15 g of agar in 1 l of distilled water) agar media [73] at 28°C for 7 days in the dark, followed by constant illumination with mycelia removed for 3 days.

### Phylogenetic tree construction

Phylogenetic tree of dynamin-related proteins and MoDnm1 homologues from several other species were drawn by the divergence distance method using the CLUSTAL_W and MEGA 5.1 programs. Neighbour-joining tree with 1000 bootstrap replicates of phylogenetic relationships. Species names and GenBank accession numbers are as follows: XP_003717217.1 (*M*. *oryzae* MoDnm1); XP_003712225.1 (*M*. *oryzae* MoVps1); XP_003708884.1 (*M*. *oryzae* MoDnm2); XP_003721138.1 (*M*. *oryzae* MoDnm3); XP_009217281.1 (*Gaeumannomyces graminis* GgDnm1); NP_013100.1 (*S*. *cerevisiae* Dnm1p); NP_542420.1 (*R*. *norvegicus* Dyn1); XP_011516636.1 (*H*. *sapiens* Drp1).

### Targeted gene deletion, amino acid substitutions and complementation

The gene deletion mutants were generated using the standard one-step gene replacement strategy. First, two 1.0 kb of sequences flanking the targeted genes were PCR amplified with primer pairs (S2 Table). Then, the resulting PCR products of *MoDNM1*, *MoDNM2*, *MoDNM3*, *MoFIS1*, and *MoMDV1* were digested with restriction endonucleases and ligated with the *HPH* cassette released from pCX62. Finally, the completed inserts were sequenced. The 3.4 kb fragments, which contain the flanking sequences and hygromycin resistance cassette, were amplified and transformed into protoplasts of Guy11. Putative mutants were screened by PCR and confirmed by Southern blot analysis. The complement fragments, which contain the entire *MoDNM1*, *MoFIS1*, *MoMDV1*, *MoDNM1* ^K43A^, *MoDNM1* ^T64G^, *MoDNM1* ^G157V^, *MoDNM1* ^D226A^, *MoDNM1* ^F627A^, *MoDNM1* ^F631A^, *MoDNM1* ^F632A^, *MoDNM1* ^ΔDYN^ and *MoDNM1* ^ΔGED^ genes with their native promoter regions, were amplified by PCR (Phanta Super-Fidelity DNA Polymerase, Vazyme Biotech Co., Nanjing, China) with primers (S2 Table) and inserted into pYF11 (bleomycin resistance) to complement the respective mutant strains.

### Assays for appressorium formation and appressorium turgor

For appressorium formation, conidia harvested from 10-day-old cultures were filtered through two-layers of miracloth and washed with double-distilled water (ddH_2_O) for three times. Droplets (20 μl) of conidial suspension (5 x 10^4^ spores/ml) were placed on cover glass (Fisher-brand, UK) and incubated at 28°C. The appressorium turgor was measured using an incipient cytorrhysis (cell collapse) assay with a 1.0 to 4.0 M glycerol solution. The water surrounding the appressoria was removed carefully and then replaced with an equal volume (20 μl) of glycerol (1.0 to 4.0 M). Appressoria were observed through direct microscopic examination and percentages were obtained from at least 100 conidia per replicate at 24 h in at least three experiments.

### Virulence assays

Virulence assays were performed as described [74]. Conidia were harvested from 10-day-old SDC agar cultures, filtered through one-layer Miracloth and resuspended by 0.2% (w/v) gelatin solution to a concentration of 5 x 10^4^ spores/ml. For detached barley assay, leaves from 7-day-old barley (*H*. *vulgare* cv. Four-arris) seedlings were drop-inoculated with three droplets (20 μl) of conidial suspension. Photographs were taken 5 days after incubation alternating light and dark at 25°C. For rice seedling spraying assay, two-week-old seedlings of rice (*O*. *sativa* cv. CO39) were sprayed with 5 ml of conidial suspension of each treatment. Inoculated plants were kept in a growth chamber at 25°C with 90% humidity and in the dark for the first 24 h, followed by a 12 h/12 h light/dark cycle. Lesion formation was daily checked and photographed 7 days after inoculation [75]. For ‘relative fungal growth’ assay, total DNA was extracted from 1.5 g disease leaves and test by qRT-PCR (HiS cript II Reverse Transcriptase, Vazyme Biotech Co., Nanjing, China) with 28S/Rubq1 primers (S2 Table) [76].

For microscopic observation of penetration and invasive hyphae expansion, conidial suspension (1 x 10^5^ spores/ml) was inoculated in the inner leaf sheath. After incubation for 30 h or 48 h at 28°C with 90% humidity, the inner leaf sheath cuticle cells were observed under Zeiss Axio Observer A1 inverted microscope.

### Observation of glycogen metabolism and lipid droplets in the germinating conidia and appressoria

The glycogen metabolism in the germinating conidia and appressoria of strains were visualized by staining these tissues with glycogen staining solution containing 60 mg/ml KI and 10 mg/ml I_2_ [77]. Once the samples become yellowish-brown, the glycogen deposits can be visualized in bright field optics with Zeiss Axio Observer A1 inverted microscope.

The lipid droplets in the germinating conidia and appressoria of strains were visualized by staining these tissues with a Nile red solution consisting of 50 mM Tris/maleate buffer (pH 7.5) and 2.5 mg/ml Nile red Oxazone (9-diethylamino-5H-benzo-a-phenoxazine-5-one, Sigma) [10, 55, 78]. After 3 min incubation, the lipid droplets in the conidia and appressoria began to fluoresce and were observed under Zeiss Axio Observer A1 inverted microscope

### Endocytosis assays

To examine endocytosis, strains were grown on a thin layer of CM agar on the microscope slides. After 40 h incubation at 28°C, the hyphae were stained with N-(3-triethylammoniumpropyl)-4-(p-diethylamino-phenyl-hexatrienyl) pyridinium dibromide (FM4-64) (Molecular Probes Inc., Eugene, OR, USA) following several times washing by ddH_2_O [79]. Photographs were taken under confocal fluorescence microscope (Zeiss LSM710, 63x oil).

### Observation of subcellular localization, peroxisomal and mitochondrial fission

To investigate the cellular localization of MoDnm1, MoMdv1, and MoFis1, three genes fused with a GFP tag in the N-terminus and a fluorescent marker appended with a type I peroxisomal targeting signal (RFP-PTS1) were co-transformed into three deletion mutants, respectively. Green and red fluorescence were observed in both vegetative hyphae grown in fluid complete medium (CM) for 24 h and conidia harvested from 10-day-old SDC medium plates under confocal fluorescence microscope (Zeiss LSM710, 63x oil). In addition, three GFP fusion proteins, including GFP-Dnm1, GFP-Mdv1, and GFP-Fis1 were expressed in three deletion mutants, respectively. Hyphae of GFP signal strains were incubated with 100 nM MitoTracker Red CMXRos (Invitrogen, Cat. M7512) for 2 min at room temperature. Green and red fluorescence were observed in both vegetative hyphae grown in fluid complete medium (CM) for 24 h and conidia which harvested from 10-day-old SDC medium plates under confocal fluorescence microscope (Zeiss LSM710, 63x oil).

To investigate peroxisomal fission, RFP-PTS1 was transformed to Guy11 and deletion mutants. For mitochondrial fission, the mitochondria localization fusion protein MoIlv2-GFP was expressed in Guy11 and deletion mutants. Red or green fluorescence was observed in vegetative hyphae grown in fluid complete medium (CM) for 24 h, conidia which harvested from 10-day-old SDC medium plates and appressoria incubated on cover glass (Fisher-brand, UK) for 8 h at 28°C.

### Transmission electron microscopy observation

Vegetative hyphae of indicated strains were cultured in fluid complete medium (CM) for 30 h and conidia were harvested from 10-day-old SDC medium plates. For Transmission electron microscopy observation, hyphae and conidia were fixed with 2.5% glutaraldehyde in phosphate buffer (pH 7.0) for more than 4 h, washed three times in the phosphate buffer, fixed with 1% OsO_4_ in phosphate buffer (pH 7.0) for 1 h and washed three times in the phosphate buffer. Then, the specimen was firstly dehydrated by a graded series of ethanol (30, 50, 70, 80, 90, 95 and 100%) for about 15 to 20 minutes at each step, transferred to absolute acetone for 20 minutes. Later, the specimen was placed in 1:1 mixture of absolute acetone and the final Spurr resin mixture for 1 h at room temperature; then transferred to 1:3 mixture of absolute acetone and the final resin mixture for 3 h and to final Spurr resin mixture for overnight. Specimen was placed in capsules contained embedding medium and heated at 70˚C for 9 h. The specimen sections were stained by uranyl acetate and alkaline lead citrate for 15 min respectively and observed under transmission electron microscopy (Hitachi H-7650).

### Inhibit or induce fission by chemical means

To inhibit peroxisomal fission, 10 mM PPARγ (nuclear receptor peroxisome proliferator activated receptor) inhibitor GW9662 (MedChem Express, HY-16578, USA) was added in conidial suspension. Similarly, 10 mM mitochondrial fission inhibitor Midvi-1 (MedChem Express, HY-15886, USA) was added in conidial suspension [59]. Conidia were incubated on cover glass (Fisher-brand, UK) at 28°C for 8 h and observed under confocal fluorescence microscope (Leica TCS SP8, 100x oil). To induce peroxisomal fission, 0.1% V/V oleate was added in conidial suspension. Similarly, 1% V/V glycerol was added to conidial suspension to induce mitochondrial fission. Conidia were incubated on cover glass (Fisher-brand, UK) at 28°C for 8 h and observed under confocal fluorescence microscope (Leica TCS SP8, 100x oil).

For microscopic observation of penetration and infectious hyphae expansion, conidial suspensions (1 x 10^5^ spores/ml) were inoculated on the back of detached barley. After incubation for 20, 24 or 26 h under humid conditions at 28°C, the barley back cuticle cells were observed under Axio Observer A1 Zeiss inverted microscope.

### Mitophagy, pexophagy and autophagy analysis

For mitophagy assays, the strains were grown in complete medium for two days, followed by 30 h growth in basal medium with 1.5% v/v glycerol. Mycelia were then starved by culturing in minimal medium lacking nitrogen for 12 h and total protein were resolved by 10% SDS-PAGE followed by Western blotting with anti-Porin antibody (mouse; 1:2,000; Invitrogen, 459500) [61, 72]. For pexophagy assays, strains were grown in CM for two days, followed by 20 h growth in basal medium with 1% v/v oleate. Mycelia were then subjected to nitrogen starvation for 12 h and total protein were resolved by 10% SDS-PAGE followed by Western blotting with anti-Pex14 antibody (rabbit; 1:2,000; Agrisera, AS08372) [61]. For autophagy assays, the GFP-Atg8 expressing strains were grown in complete medium (CM) for 30 h, then washed and subjected to nitrogen starvation (cultured in MM-N for 2 or 5 h) to induce nonselective autophagy. Mycelia induced for 2 or 5 h was microscopy observation and biochemical assays for GFP-Atg8 cleavage. Mycelia induced for 5 h were analyzed using Transmission electron microscopy. Immunobloting for GFP-Atg8 cleavage was done with anti-GFP (mouse; 1:5000; Abmart) and anti-*β*-Actin antibodies (mouse; 1:5000; zoonbio, ABM-0001) [80]. The amount of free GFP and GFP-Atg8 were conducted by densitometric analysis (Image-pro plus, Media Cybernetics Inc., Shanghai, China).

### Yeast two-hybrid assays

The bait constructs were generated by cloning *MoDNM1* and *MoFIS1* full-length cDNAs into pGBKT7, respectively. The cDNAs of *MoFIS1* and *MoMDV1* were cloned into pGADT7 as the prey constructs (see primers in S2 Table). The resulting prey and bait constructs were confirmed by sequencing analysis and transformed in pairs into yeast strain AH109 as the description of BD library construction & screening kit (Clontech, USA). The Trp^+^ and Leu^+^ transformants were isolated and assayed for growth on SD-Trp-Leu-His-Ade medium and the expression of LacZ reporter gene following the instructions provided by Clontech. Yeast stains for positive and negative controls were provide by the BD library construction & screening kit.

### GST pull down assays

For protein production in *Escherichia coli*, the full length of *MoDNM1* and *MoFIS1* was inserted into the pGEX4T-2 vector and the full length of *MoDNM1* and *MoFIS1* was inserted into the pET32a vector. The resultant plasmid DNA was transformed into Rosetta 2 (DE3) cells (Novagen, Madison, WI). Protein expression was induced for 8 h at 25°C after the addition of isopropy-*β*-D-thiogalactoside (IPTG) to a final concentration of 0.1 mM. Cells were collected by centrifugation, washed, and stored at -70°C. To extract proteins, cells were suspended in lysis buffer containing 0.5 mM EDTA, 1% Triton, 20 mM Tris-HCl, 0.15 M NaCl, 1 mM DTT and protease inhibitors (1 mM PMSF) and ultrasonic wall-breaking under frequency: 100–200 w 2 min, pause 4 s run 2 s. Samples were centrifuged at 13,000 rpm for 15 min at 4°C and the supernatants were collected. For protein purification, supernatants were mixed with glutathione-Sepharose resin (Amersham Pharmacia, Piscataway, NJ) for 2 h, centrifuged at 500 rpm for 2 min at 4°C, washed with Tris-NaCl buffer (1 mM PMSF, 1% Triton, 50 mM Tris-HCl, 100 mM NaCl) and the proteins were eluted with 15 mM glutathione. Proteins were also verified by SDS-PAGE analysis and Western blotting with the anti-GST antibody (Abmart). For binding, 500 μl of the GST- proteins extract were added to glutathione-Sepharose resin washed with Tris-NaCl buffer. The proteins His- supernatants were added to resin and incubated overnight with gentle rotation at 4°C, precipitated, and washed three times with Tris-NaCl buffer. SDS-containing gel loading buffer (100 μl) was added to the resin before a brief boiling. Samples (4 μl) were analyzed by SDS-PAGE electrophoresis and Western blotting using anti-His and anti-GST antibodies. GST protein was used as a control.

## Supporting Information

S1 Fig Southern blot analysis of deletion mutants. (A) Strategy of knocking out target genes in *M*. *oryzae* genome. Thin lines below the arrows indicate the probe sequence of each gene. (B and C) Southern blot analysis of deletion mutants with gene specific probes (probe 1, 3, 4, 5 and 6) and hygromycin phosphotransferase (*HPH*) probe (probe 2). EI: *EcoR* I, EV: *EcoR* V.
(TIF)

S2 Fig MoDnm1, MoMdv1, and MoFis1 are involved in appressorium turgor. Statistical analysis of collapsed appressoria numbers on hydrophobic surface after 24 h incubation. Error bars represent the standard deviations and asterisks represent significant differences (Duncan's new multiple range test, *p*<0.01).
(TIF)

S3 Fig MoDnm1, MoMdv1, and MoFis1 have no effect on mobilization of glycogen and lipid during appressorium morphogenesis. (A and C) Conidia from different strains were germinated on hydrophobic plastic cover slips. Samples were removed at 0, 2, 4, 6, 8, 12 h and stained for the presence of glycogen with iodine solution. Yellowish-brown glycogen deposits were observed under Axio Observer A1 Zeiss inverted microscope. Bar = 5 μm. (B and D) Conidia from different strains were germinated on hydrophobic plastic cover slips. Samples were removed at 0, 2, 4, 6 and 8 h and stained for the presence of lipid with Nile red solution. Lipid bodies were observed under Axio Observer A1 Zeiss inverted microscope. Bar = 5 μm.
(TIF)

S4 Fig Phenotypes of double deletion mutants. (A and B) Strategy of knocking out target genes in *M*. *oryzae* genome. (C) PCR analysis of gene knockout mutants with gene specific probes, hygromycin phosphotransferase (*HPH*) and bleomycin probe (*BLE*). (D) Seven-day-old cultures of different strains on CM plates. ±SD was calculated from three repeated experiments and asterisks indicate statistically significant differences (Duncan's new multiple range test, *p<0*.*01*). (E) Statistical analysis of the conidial length of the indicated strains. ±SD was calculated from three repeated experiments.
(TIF)

S5 Fig Impaired peroxisomal and mitochondrial fission result in pathogenicity reduction. (A to D) Observation and statistical analysis of the peroxisomes (C and D mitochondria) in appressoria of different indicated strains. All strains were incubated with peroxisomal fission inducer (0.1% V/V oleate) or mitochondrial fission inducer (1% V/V glycerol). Arrows denote tubular peroxisomes or mitochondria; Asterisks denote statistical significances (Duncan's new multiple range test, *p*<0.01). Bar = 5 μm. (E and F) detailed observation and statistics for infectious growth in detached barley cells at 20 or 26 hpi. Appressorium penetration sites (n = 100) were observed and counted by rating the invasive hyphae from type 1 to 4. The experiment was repeated three times. Error bars represent ±SD. Arrows denote appressorium (AP) and invasive hyphae (IH). Bar = 5 μm.
(TIF)

S6 Fig MoDnm1, MoMdv1, and MoFis1 have no effect on ATP production. (A) Detached barley was drop-inoculated with conidial suspensions with exogenous ATP and examined 24 hpi by fluorescent microscopy (Zeiss Axio Observer A1 20x). Arrows denote appressorium (AP) and invasive hyphae (IH). (B) ATP content in mycelia of indicated strains is tested by HPLC analysis.
(TIF)

S7 Fig Structure and motif prediction of MoDnm1. (A) Alignment of Dnm1 proteins from different organisms. Identical and similar amino acid residues are outlined in black and gray, respectively. The asterisks indicate the position of the *MoDNM1* ^K43A^, *MoDNM1* ^T64G^, *MoDNM1* ^G157V^, *MoDNM1* ^D226A^ mutations. (B) Schematic diagram of the domains and important motifs in MoDnm1. (C) Alignment of a segment of InsB from Dnm1 homologues. The asterisks mark the position of *MoDNM1* ^F627A^, *MoDNM1* ^F631A^, *MoDNM1* ^F632A^ mutations. (D) Western blot analysis of MoDnm1 in motif mutation or domain deletion strains with anti-GFP antibody.
(TIF)

S8 Fig The InsB motif, DYNc and GED domains of MoDnm1 are important for Growth and virulence. (A) Seven-day-old cultures of different strains on CM plates. Take statistical analysis of the colony diameter of the indicated strains. ±SD was calculated from three repeated experiments and asterisks indicate statistically significant differences (Duncan's new multiple range test, *p*<0.01). (B) Diseased rice leaves were collected after 7 d inoculation. Total DNA was extracted from per 1.5 g disease leaves and test by qRT-PCR (HiS cript II Reverse Transcriptase, Vazyme Biotech Co., Nanjing, China) with 28S/Rubq1 primers. Different letters indicate statistically significant differences (Duncan's new multiple range test, *p*<0.01).
(TIF)

S9 Fig Dissection of MoFis1 domain functions. (A) Seven-day-old cultures of different strains on CM plates. (B) Rice seedlings spraying assay with conidial suspensions and examined at 7 dpi. (C) Three N-terminal truncated MoFis1 constructs MoFis1^Δ1–29^ (Δ1–29), MoFis1^Δ1–57^ (Δ1–57) and MoFis1^Δ1–88^ (Δ1–88) were co-expressed with RFP-PTS1, respectively. RFP signals were observed and counted by Image-pro plus software. Asterisks indicate statistically significant differences (Duncan's new multiple range test, *p*<0.01). Error bars represent ±SD. (D) Conidia of GFP-MoFis1, GFP-MoFis1^Δ148–155^ (Δ148–155) and GFP-MoFis1^Δ127–155^ (Δ127–155) mutants were observed under confocal microscopy (Leica TCS SP8, 100x oil). Strong green and red signals were observed in these three mutants.
(TIF)

S1 Table Comparison of mycological characteristics among strains. (DOCX)

S2 Table Primers used in this study. (DOCX)
