# Supplementary figures and images for: MoDnm1 Dynamin Mediating Peroxisomal and Mitochondrial Fission in Complex with MoFis1 and MoMdv1 Is Important for Development of Functional Appressorium in Magnaporthe oryzae
Source: PLoS Pathog. 2016 Aug 24;12(8):e1005823. doi: 10.1371/journal.ppat.1005823 (PMC4996533; doi:10.1371/journal.ppat.1005823)

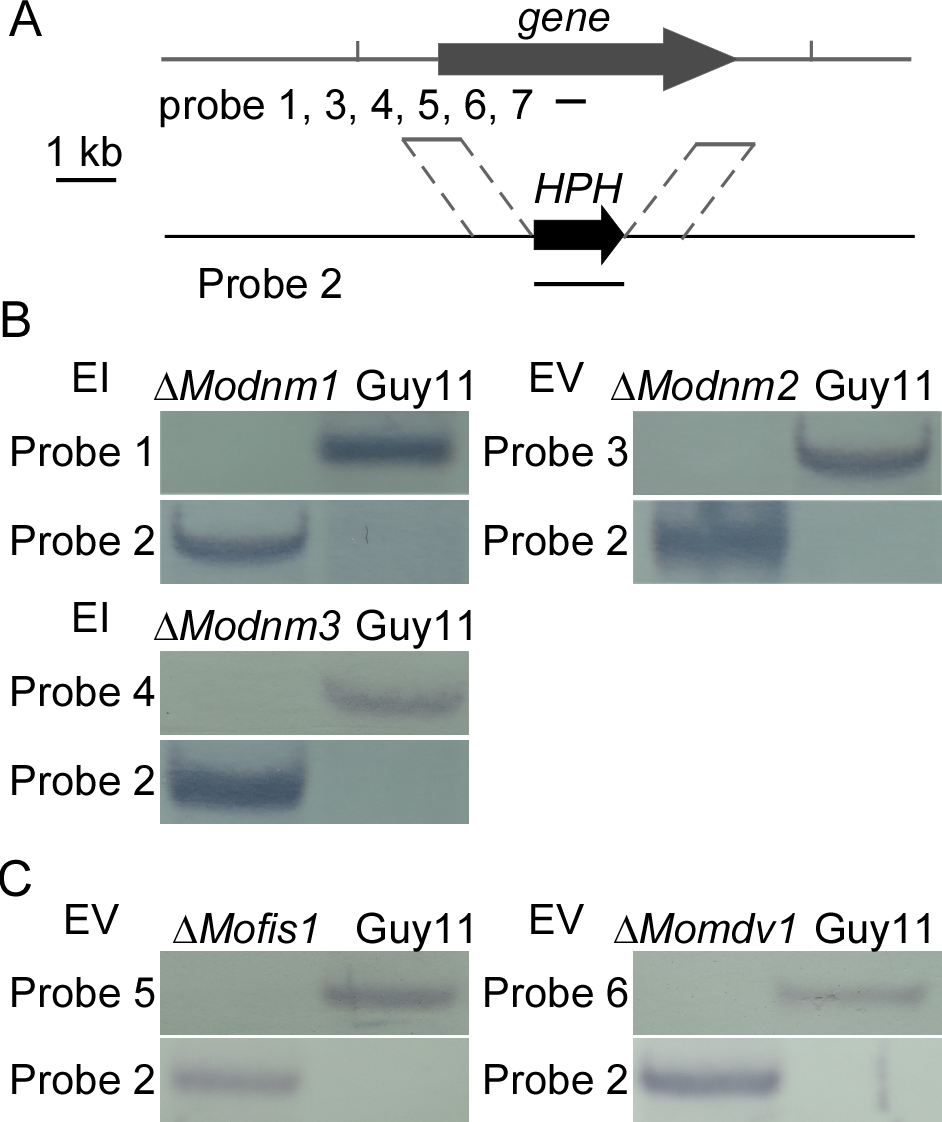

Supplement: S1 Fig — (A) Strategy of knocking out target genes in M. oryzae genome. Thin lines below the arrows indicate the probe sequence of each gene. (B and C) Southern blot analysis of deletion mutants with gene specific probes (probe 1, 3, 4, 5 and 6) and hygromycin phosphotransferase (HPH) probe (probe 2). EI: EcoR I, EV: EcoR V. (TIF) [file ppat.1005823.s001.tif]

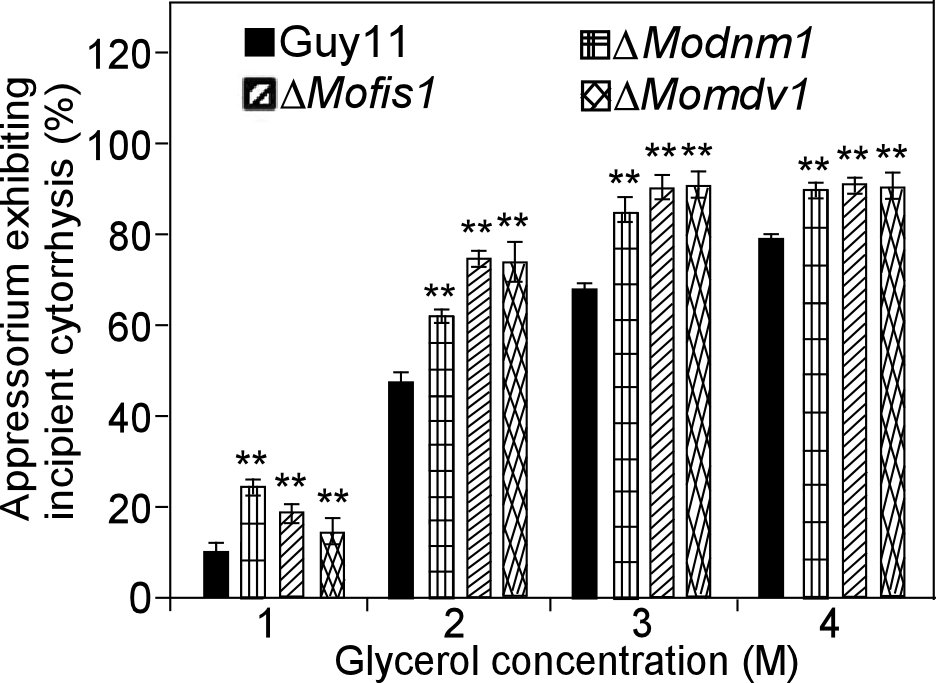

Supplement: S2 Fig — Statistical analysis of collapsed appressoria numbers on hydrophobic surface after 24 h incubation. Error bars represent the standard deviations and asterisks represent significant differences (Duncan's new multiple range test, p<0.01). (TIF) [file ppat.1005823.s002.tif]

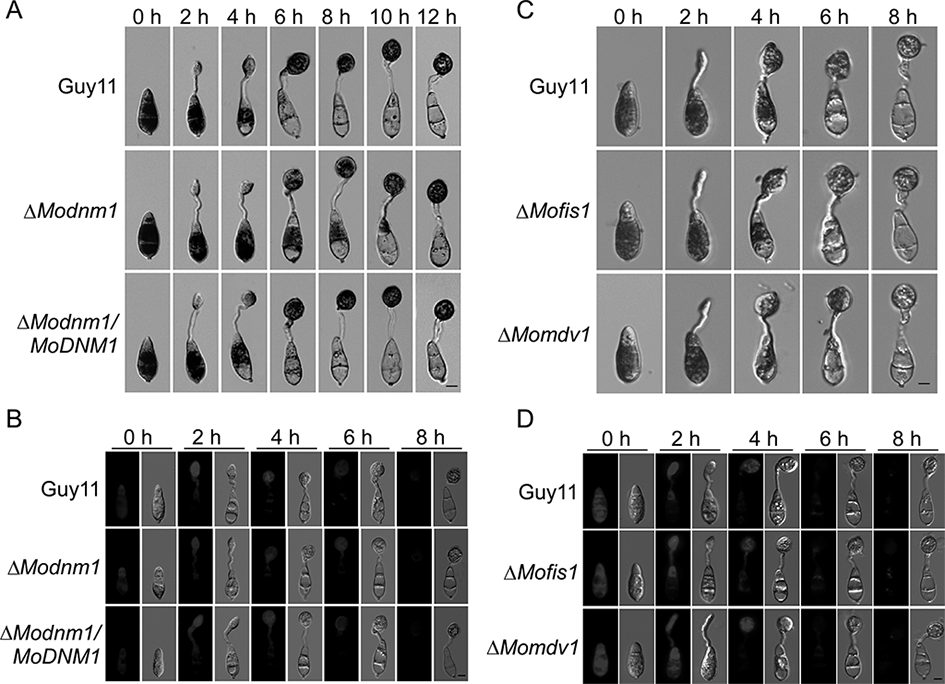

Supplement: S3 Fig — (A and C) Conidia from different strains were germinated on hydrophobic plastic cover slips. Samples were removed at 0, 2, 4, 6, 8, 12 h and stained for the presence of glycogen with iodine solution. Yellowish-brown glycogen deposits were observed under Axio Observer A1 Zeiss inverted microscope. Bar = 5 μm. (B and D) Conidia from different strains were germinated on hydrophobic plastic cover slips. Samples were removed at 0, 2, 4, 6 and 8 h and stained for the presence of lipid with Nile red solution. Lipid bodies were observed under Axio Observer A1 Zeiss inverted microscope. Bar = 5 μm. (TIF) [file ppat.1005823.s003.tif]

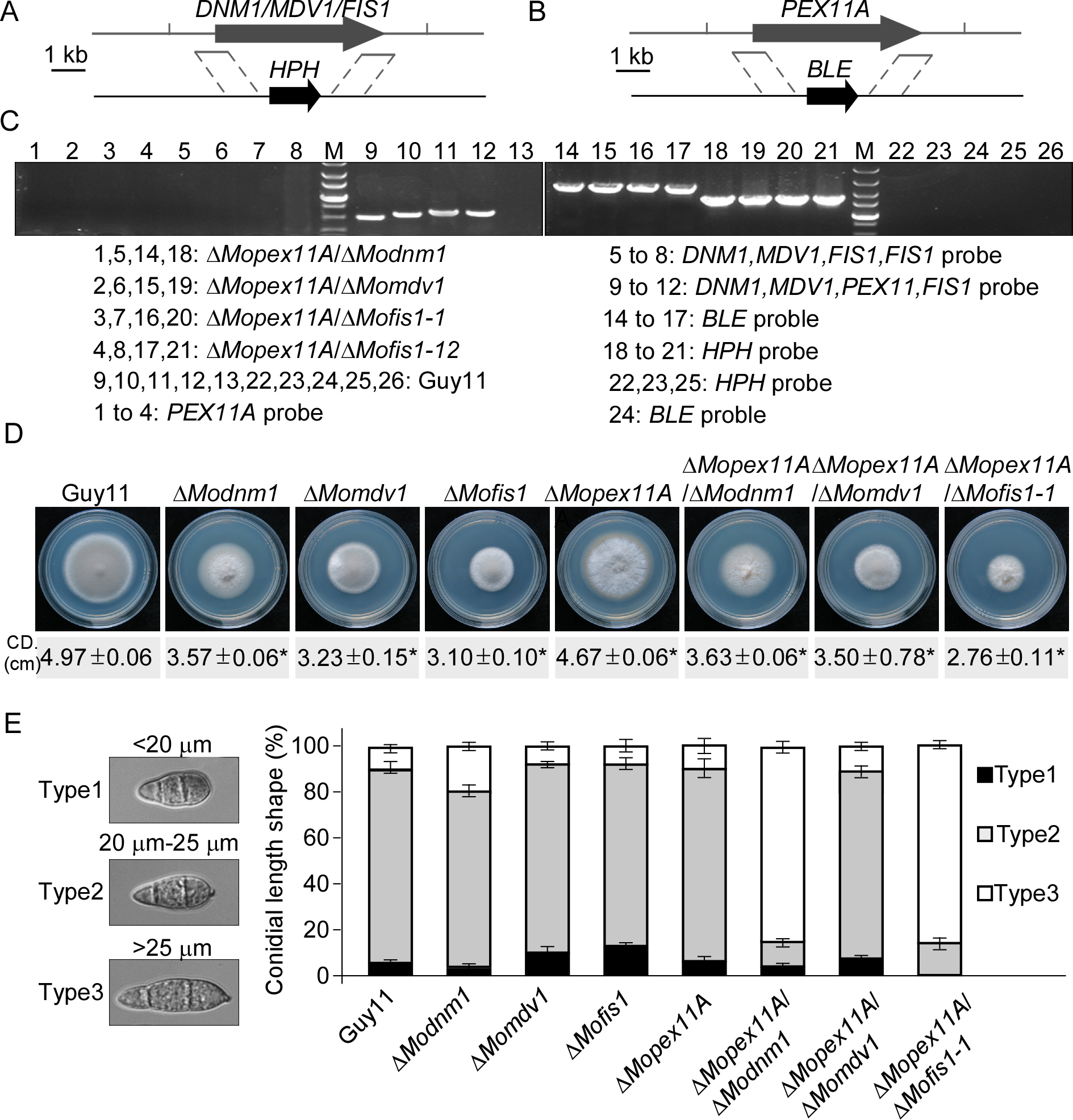

Supplement: S4 Fig — (A and B) Strategy of knocking out target genes in M. oryzae genome. (C) PCR analysis of gene knockout mutants with gene specific probes, hygromycin phosphotransferase (HPH) and bleomycin probe (BLE). (D) Seven-day-old cultures of different strains on CM plates. ±SD was calculated from three repeated experiments and asterisks indicate statistically significant differences (Duncan's new multiple range test, p<0.01). (E) Statistical analysis of the conidial length of the indicated strains. ±SD was calculated from three repeated experiments. (TIF) [file ppat.1005823.s004.tif]

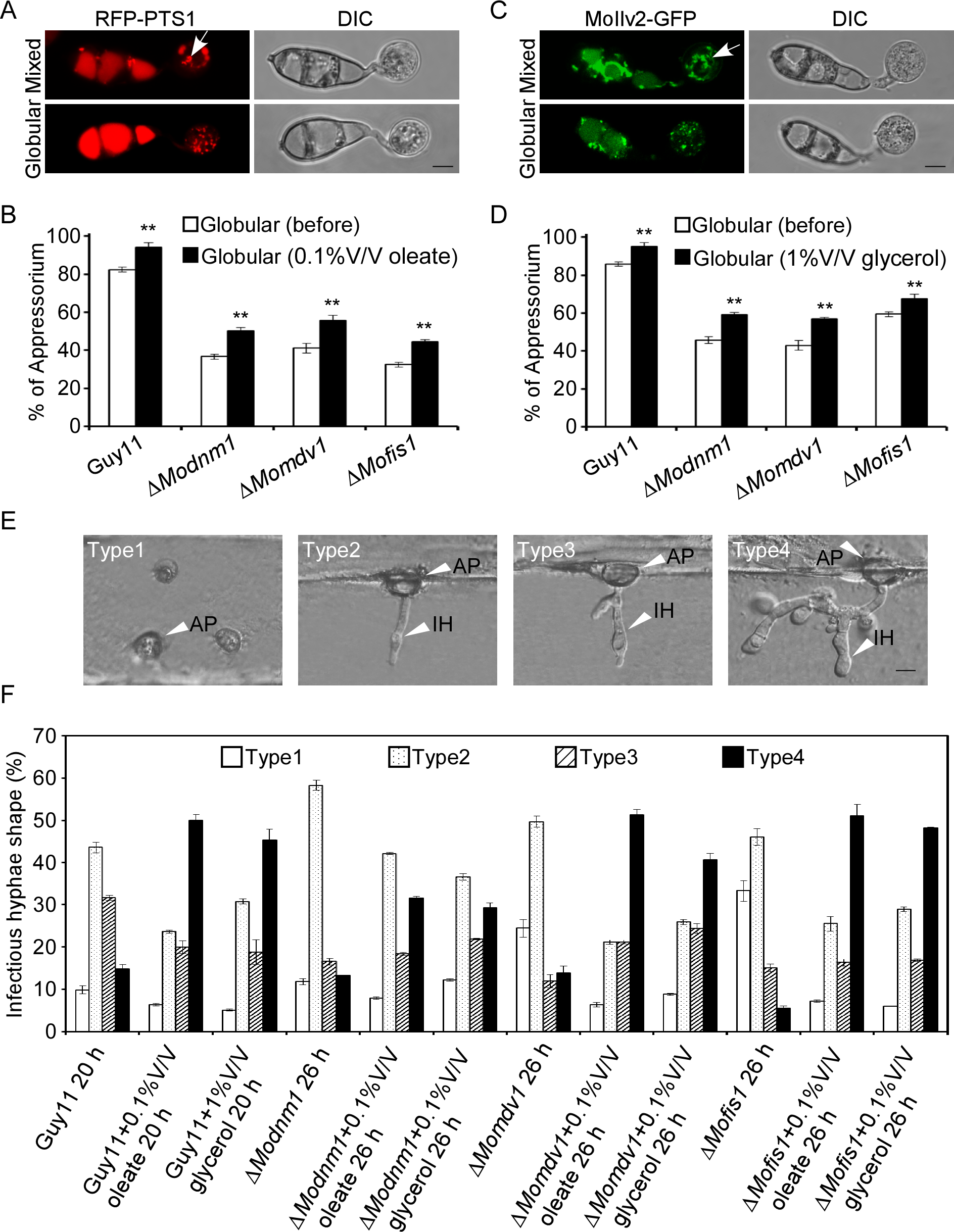

Supplement: S5 Fig — (A to D) Observation and statistical analysis of the peroxisomes (C and D mitochondria) in appressoria of different indicated strains. All strains were incubated with peroxisomal fission inducer (0.1% V/V oleate) or mitochondrial fission inducer (1% V/V glycerol). Arrows denote tubular peroxisomes or mitochondria; Asterisks denote statistical significances (Duncan's new multiple range test, p<0.01). Bar = 5 μm. (E and F) detailed observation and statistics for infectious growth in detached barley cells at 20 or 26 hpi. Appressorium penetration sites (n = 100) were observed and counted by rating the invasive hyphae from type 1 to 4. The experiment was repeated three times. Error bars represent ±SD. Arrows denote appressorium (AP) and invasive hyphae (IH). Bar = 5 μm. (TIF) [file ppat.1005823.s005.tif]

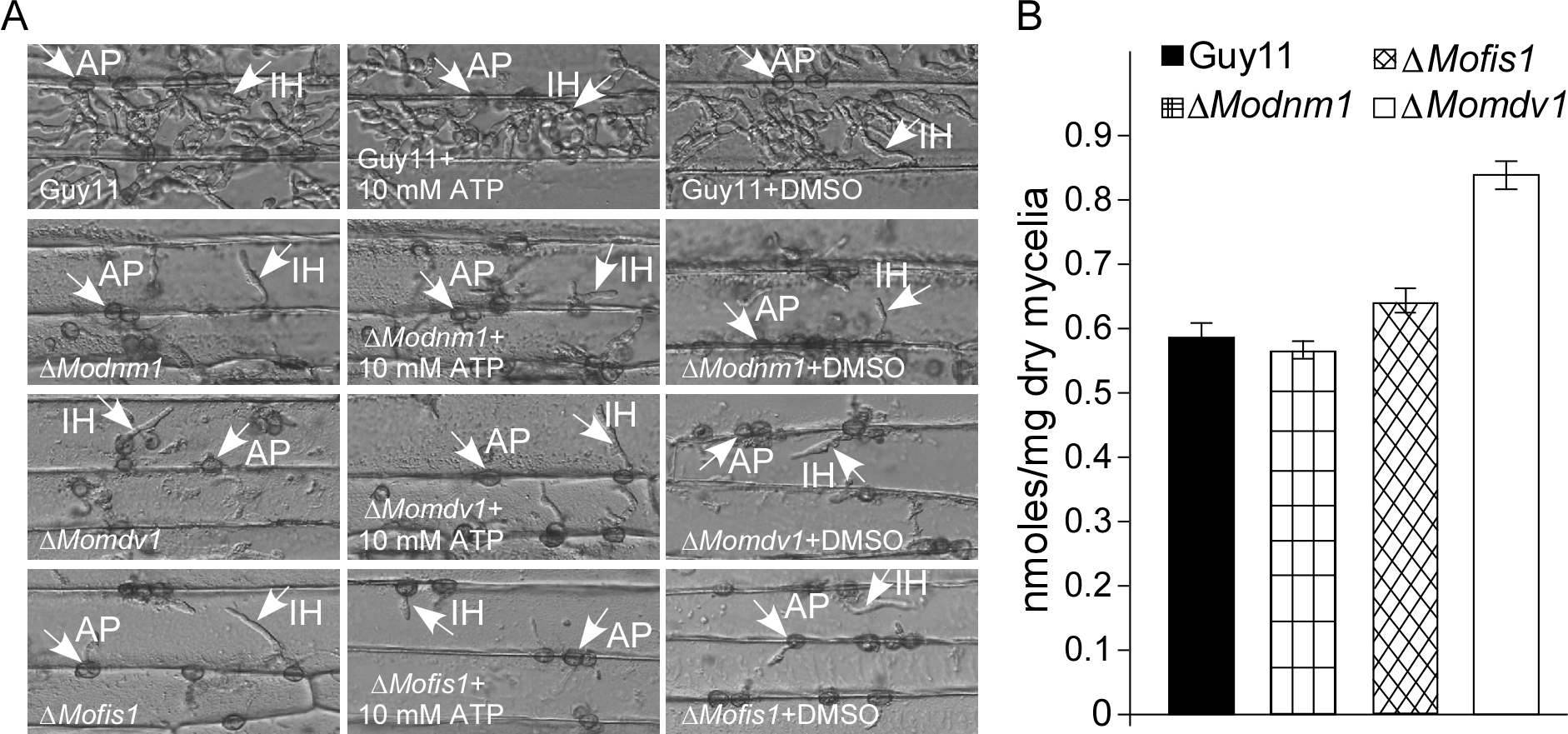

Supplement: S6 Fig — (A) Detached barley was drop-inoculated with conidial suspensions with exogenous ATP and examined 24 hpi by fluorescent microscopy (Zeiss Axio Observer A1 20x). Arrows denote appressorium (AP) and invasive hyphae (IH). (B) ATP content in mycelia of indicated strains is tested by HPLC analysis. (TIF) [file ppat.1005823.s006.tif]

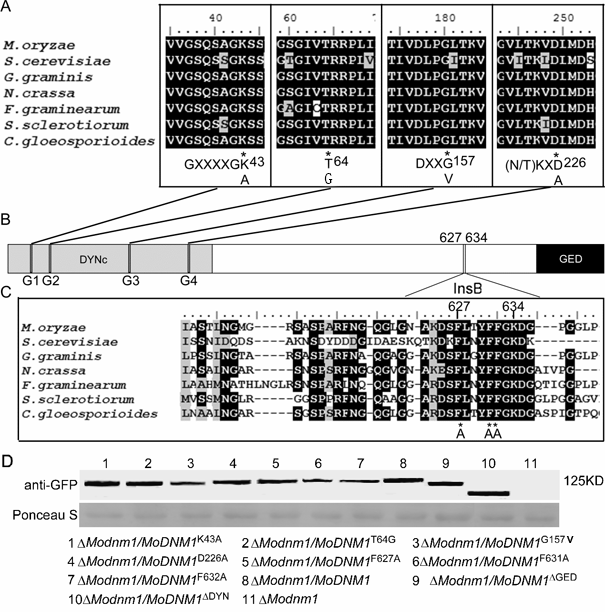

Supplement: S7 Fig — (A) Alignment of Dnm1 proteins from different organisms. Identical and similar amino acid residues are outlined in black and gray, respectively. The asterisks indicate the position of the MoDNM1 K43A, MoDNM1 T64G, MoDNM1 G157V, MoDNM1 D226A mutations. (B) Schematic diagram of the domains and important motifs in MoDnm1. (C) Alignment of a segment of InsB from Dnm1 homologues. The asterisks mark the position of MoDNM1 F627A, MoDNM1 F631A, MoDNM1 F632A mutations. (D) Western blot analysis of MoDnm1 in motif mutation or domain deletion strains with anti-GFP antibody. (TIF) [file ppat.1005823.s007.tif]

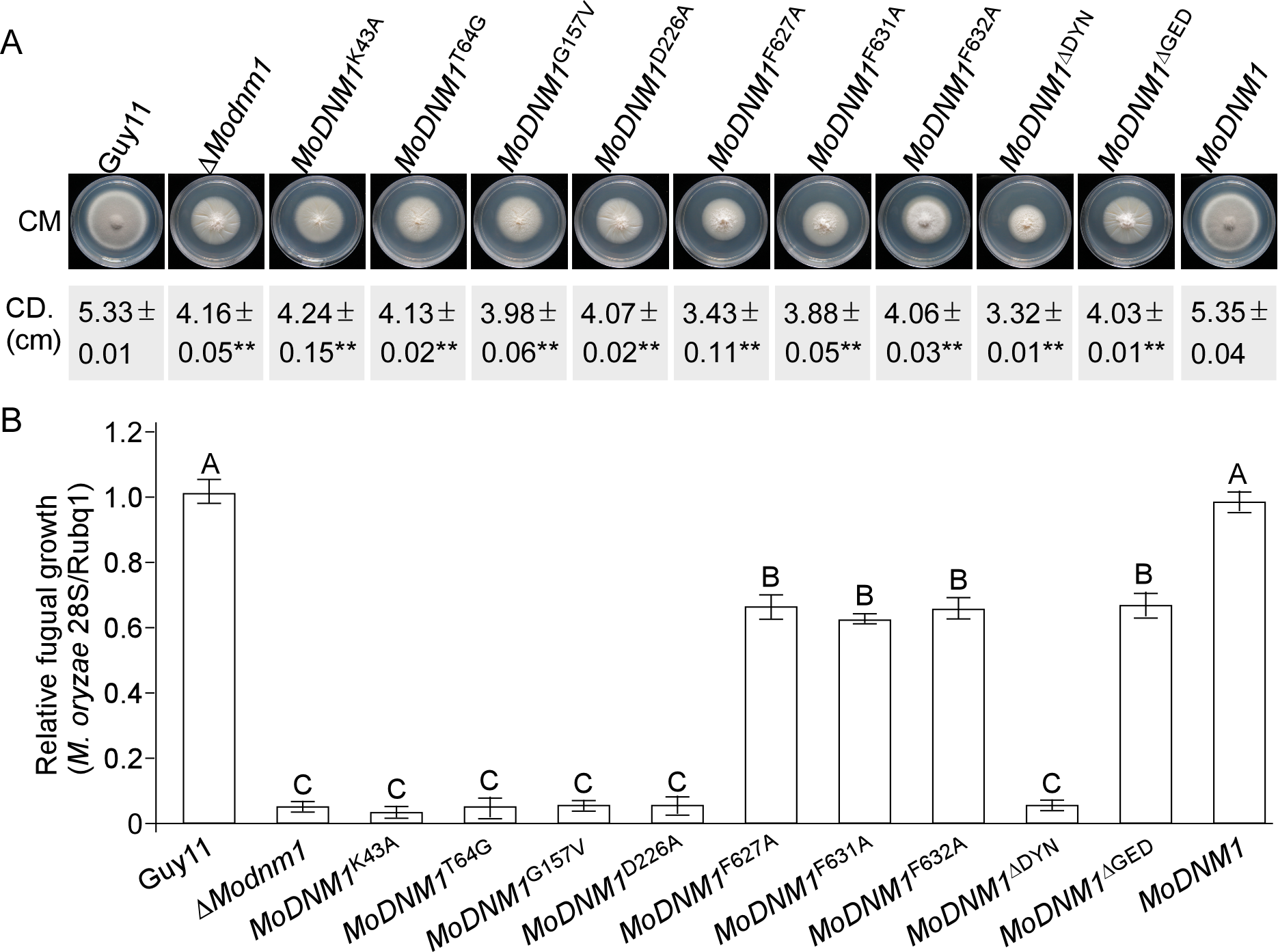

Supplement: S8 Fig — (A) Seven-day-old cultures of different strains on CM plates. Take statistical analysis of the colony diameter of the indicated strains. ±SD was calculated from three repeated experiments and asterisks indicate statistically significant differences (Duncan's new multiple range test, p<0.01). (B) Diseased rice leaves were collected after 7 d inoculation. Total DNA was extracted from per 1.5 g disease leaves and test by qRT-PCR (HiS cript II Reverse Transcriptase, Vazyme Biotech Co., Nanjing, China) with 28S/Rubq1 primers. Different letters indicate statistically significant differences (Duncan's new multiple range test, p<0.01). (TIF) [file ppat.1005823.s008.tif]

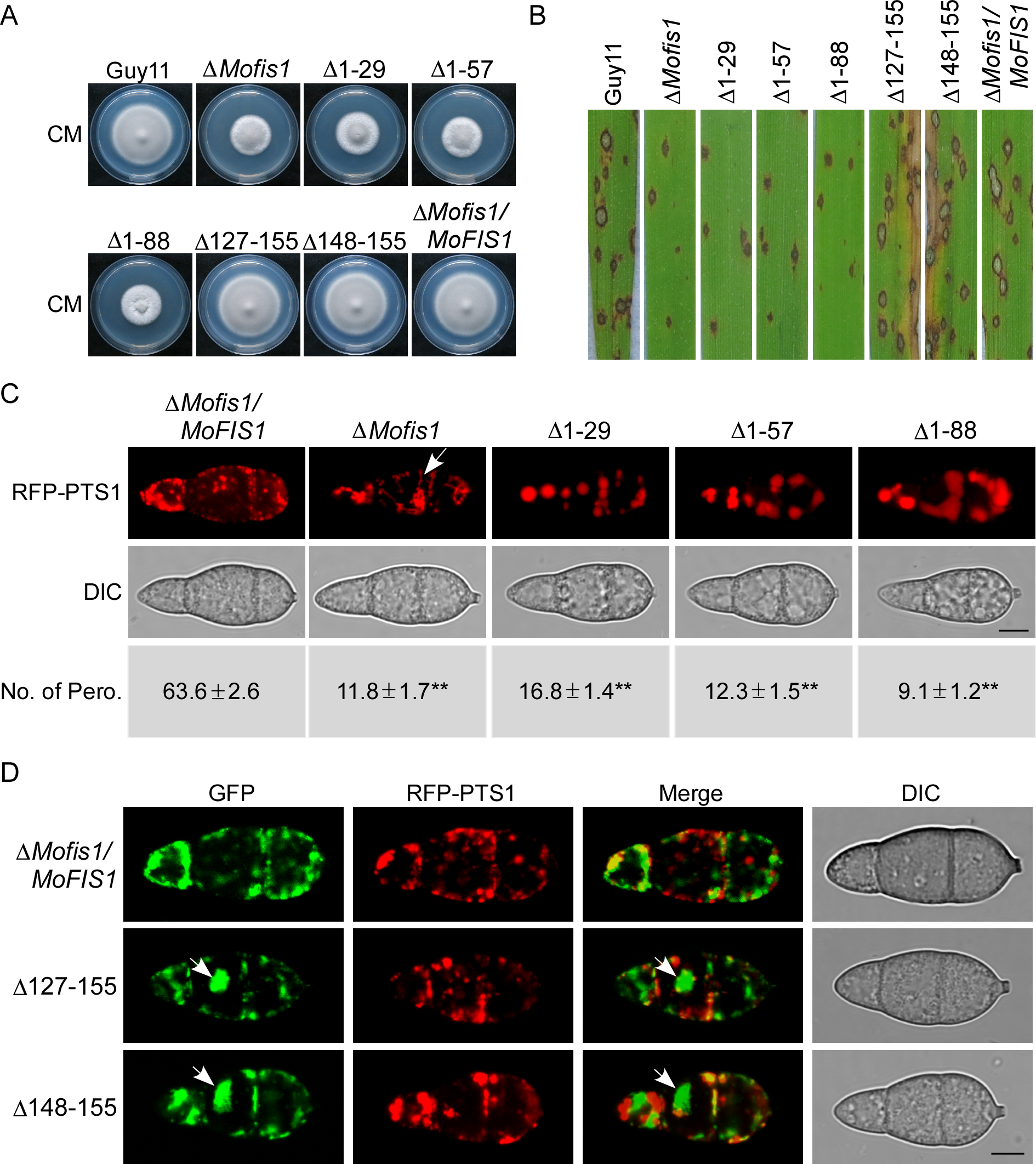

Supplement: S9 Fig — (A) Seven-day-old cultures of different strains on CM plates. (B) Rice seedlings spraying assay with conidial suspensions and examined at 7 dpi. (C) Three N-terminal truncated MoFis1 constructs MoFis1Δ1–29 (Δ1–29), MoFis1Δ1–57 (Δ1–57) and MoFis1Δ1–88 (Δ1–88) were co-expressed with RFP-PTS1, respectively. RFP signals were observed and counted by Image-pro plus software. Asterisks indicate statistically significant differences (Duncan's new multiple range test, p<0.01). Error bars represent ±SD. (D) Conidia of GFP-MoFis1, GFP-MoFis1Δ148–155 (Δ148–155) and GFP-MoFis1Δ127–155 (Δ127–155) mutants were observed under confocal microscopy (Leica TCS SP8, 100x oil). Strong green and red signals were observed in these three mutants. (TIF) [file ppat.1005823.s009.tif]
