# Supplementary material for: MoDnm1 Dynamin Mediating Peroxisomal and Mitochondrial Fission in Complex with MoFis1 and MoMdv1 Is Important for Development of Functional Appressorium in Magnaporthe oryzae
Source: PLoS Pathog. 2016 Aug 24;12(8):e1005823. doi: 10.1371/journal.ppat.1005823 (PMC4996533; doi:10.1371/journal.ppat.1005823)
Supplement: S1 Table — (DOCX) [file ppat.1005823.s010.docx]

**S1 Table. Comparison of mycological characteristics among strains**

| **Strain** | **Mycelial Growth (cm)** | | **Conidiation**^a^  **(x10^4^/cm^2^)** |
| --- | --- | --- | --- |
|  | **CM** | **MM** |  |
| **Guy11** | 5.13±0.1A | 4.56±0.5A | 13.6±0.5A |
| **Δ*Modnm1*** | 3.61±0.1B | 2.42±0.1B | 3.8±0.4B |
| **Δ*Modnm2*** | 5.11±0.1A | 4.52±0.1A | 14.6±0.8A |
| **Δ*Modnm3*** | 5.24±0.3A | 4.61±0.2A | 13.4±0.4A |

a. Quantification of the conidial production of the indicated strains

from SDC cultures.
