# Supplementary material for: MoDnm1 Dynamin Mediating Peroxisomal and Mitochondrial Fission in Complex with MoFis1 and MoMdv1 Is Important for Development of Functional Appressorium in Magnaporthe oryzae
Source: PLoS Pathog. 2016 Aug 24;12(8):e1005823. doi: 10.1371/journal.ppat.1005823 (PMC4996533; doi:10.1371/journal.ppat.1005823)
Supplement: S2 Table — (DOCX) [file ppat.1005823.s011.docx]

**S2 Table. Primers used in this study**

| Primer | Sequence (5’-3’) | Application |
| --- | --- | --- |
| MoDnm1-F1 | GTCGACGAAAGCACTCATGGCAACTG | Amplify *MoDNM1* 5’ flank sequence, for gene knock out |
| MoDnm1-R1 | GATATCCTCTCTCTCTTTATGGCGGC |  |
| MoDnm1-F2 | TCTAGACAGATACAGGTTGCCCAGAT | Amplify *MoDNM1* 3’ flank sequence, for gene knock out |
| MoDnm1-R2 | GAGCTCGACCGGTATATCCCGAAAGC |  |
| MoDnm1-IF | ACAAGGGTATCAACCGACAG | Amplify *MoDNM1* gene probe, for southern blot and transformants screen |
| MoDnm1-IR | ATGTCCTGCTGCGATCGGTT |  |
| MoDnm1-OF | CTCGCCATCGTGATCTAGAG | Transformants screen |
| HPH ConR | GCTGATCTGACCAGTTGCCTA |  |
| MoDnm1-ComF1 | ACTCACTATAGGGCGAATTGGGTACTCAAATTGGTTCAGGTACGTCCATTGATGAG | *MoDNM1* complementation |
| MoDnm1-ComR1 | GGCGAAAATCGGTTCCGTGGTCTA |  |
| MoDnm1-ComF2 | TCTCTTTGTAGACCACGGAACCGATTTTCGCCATGGTGAGCAAGGGCGAGGA |  |
| MoDnm1-ComR2 | CTTGTACAGCTCGTCCATGC |  |
| MoDnm1-ComF3 | ATCACTCTCGGCATGGACGAGCTGTACAAGATGGCCGCCCTCGGAGACGATTTGTT |  |
| MoDnm1-ComR3 | TTACTTGTACAGCTCGTCCATGCCGAGAGTGATCTCATACCACCTCTGAGATGAT |  |
| BD- MoDnm1-F | TAATAACATATGATGGCCGCCCTCGGAGACGATT | Construction of *pGBKT7-MoDNM1* |
| BD- MoDnm1-R | TAATAAGAATTCTCATACCACCTCTGAGATGAT |  |
| GST- MoDnm1-F | TAATAAGAATTCCCATGGCCGCCCTCGGAGACGATTT | Construction of *GST-MoDNM1* |
| GST- MoDnm1-R | TAATAAGCGGCCGCTCATACCACCTCTGAGATGAT |  |
| Dnm1K43A-F1 | ACTCACTATAGGGCGAATTGGGTACTCAAATTGGTTCAGGTACGTCCATTGATGAG | Construction of *MoDNM1^K43A^*  (Dnm1K43A-F1 and Dnm1K43A-R2 are used in construction of other point mutations) |
| Dnm1K43A-R1 | TCCGGCTGACTGGGATCCAAC |  |
| Dnm1K43A-F2 | AGGTTGTCGTTGGATCCCAGTCAGCCGGAGCGTCTTCGGTCCTCGAGAACAT |  |
| Dnm1K43A-R2 | CACCACCCCGGTGAACAGCTCCTCGCCCTTGCTCACTACCACCTCTGAGATGAT |  |
| Dnm1T64G-R1 | AACAATCCCACTGCCACGGGGCA | Construction of *MoDNM1^T64G^* |
| Dnm1T64G-F2 | ATTTTCTGCCCCGTGGCAGTGGGATTGTTGGAAGGCGACCTCTGATTCTCCAGCT |  |
| Dnm1G157A-R1 | GGGCAAATCCACAAGGGTCAGGTT | Construction of *MoDNM1^G157A^* |
| Dnm1G157A-F2 | ACATGTTCTCAACCTGACCCTTGTGGATTTGCCCGTATTAACAAAGGTATGTGCTGTGCCCTG |  |
| Dnm1D226A-R1 | GACTTTGGTAAGAACACCG | Construction of *MoDNM1^D226A^* |
| Dnm1D226A-F2 | TGGGAAGGAGGACTATCGGTGTTCTTACCAAAGTCGCTCTTATGGACCATGGCACCAATG |  |
|  |  | Construction of *MoDNM1^F627A^* |
| Dnm1F627A-R1 | CGAATCCTTGGCGTTGCCTAA |  |
| Dnm1F627A-F2 | AGGGCTTAGGCAACGCCAAGGATTCGGCTTTGACGTACTTTTTTGGCAAGGA |  |
| Dnm1F631A-R1 | GTACGTCAAAAACGAATCCTTGGCG | Construction of *MoDNM1^F631A^* |
| Dnm1F631A-F2 | AGGCAACGCCAAGGATTCGTTTTTGACGTACGCTTTTGGCAAGGACGGACC |  |
|  |  |  |
| Dnm1F632A-R1 | AAAGTACGTCAAAAACGAATCCTTGGCG | Construction of *MoDNM1^F632A^* |
| Dnm1F632A-F2 | CGCCAAGGATTCGTTTTTGACGTACTTTGCTGGCAAGGACGGACCCGGA |  |
| MoDNM1^ΔDYN^-R1 | GAGGGCGGCCATGGCGAAAATCGGTT | Construction of *MoDNM1*^ΔDYN^ |
| MoDNM1^ΔDYN^-F2 | ACGGAACCGATTTTCGCCATGGCCGCCCTCAAGCCCATGGAAGATGCGCTTA |  |
| MoDNM1^ΔGED^-F | ACTCACTATAGGGCGAATTGGGTACTCAAATTGGTTCAGGTACGTCCATTGATGAG | Construction of *MoDNM1*^ΔGED^ |
| MoDNM1^ΔGED^-R | CACCACCCCGGTGAACAGCTCCTCGCCCTTGCTCACGTCAGTCAACGCCGGCTCTCCACT |  |
| MoDnm2-F1 | TAACTCGAG CAAGTATCGACATCCGTGTC | Amplify *MoDNM2* 5’ flank sequence, for gene knock out |
| MoDnm2-R1 | TAAGAATTCGCGTAAATGTCTTCAGGGTAGG |  |
| MoDnm2-F2 | TAAACTAGTCTAGTCTAGTTCTAGGTTGCCG | Amplify *MoDNM2* 3’ flank sequence, for gene knock out |
| MoDnm2-R2 | TAACCGCGGGCTTCGAGTGCACGATTGCTTTTC |  |
| MoDnm2-IF | CAAAAAGCTCAGGTCCGAAG | Amplify *MoDNM2* gene probe, for southern blot and transformants screen |
| MoDnm2-IR | GCTCTTGACTAGATCGGTCAAG |  |
| MoDnm2-OF | GCATTCTGGCAGGTCTAACTAC | Transformants screen |
| MoDnm3-F1 | TAAAAGCTTAGCAAAGTCTGAGTCGTGGAG | Amplify *MoDNM3* 5’ flank sequence, for gene knock out |
| MoDnm3-R | TAAGAATTCGGCAGTGATTGTGTCGTGTATAGC |  |
| MoDnm3-F2 | TAAACTAGTCTGATCCGAGGCCTTGAGTACAA | Amplify *MoDNM3* 3’ flank sequence, for gene knock out |
| MoDnm3-R2 | TAAGAGCTCCTGCATCGATGTGCTTGGAGATC |  |
| MoDnm3-IF | GTGATACAGTACCCAAGCAG | Amplify *MoDNM3* gene probe, for southern blot and transformants screen |
| MoDnm3-IR | CTCTGCAACCTTGACCTCAAG |  |
| MoDnm3-OF | GACAGCACAATGCTAGCCTC | Transformants screen |
| MoFis1-F1 | GACTTCTGTACACCTTGAGTCTC | Amplify *MoFIS1* 5’ flank sequence, for gene knock out |
| MoFis1-R1 | TTTTGATGATATGCCTGATGTCTAG |  |
| MoFis1-F2 | CTAGACATCAGGCATATCATCAAAA GATATCTGTCGGGGCATAACGTTGCTAA | Amplify *MoFIS1* 3’ flank sequence, for gene knock out |
| MoFis1-R2 | TGGGCAGAGAGGCTGAAATGATGA |  |
| MoFis1-IF | TCAACAGATGCTATCGATGCG | Amplify *MoFIS1* gene probe, for southern blot and transformants screen |
| MoFis1-IR | CAACTGCAACGCCACCAATGAT |  |
| MoFis1-OF | CTATGTTGGGTCATGGCCTTTC | Transformants screen |
| MoMdv1-F1 | ATATCATGCAAGCGAAAAAGG | Amplify *MoMDV1* flank sequence, for gene knock out |
| MoMdv1-R1 | GAAGAGTAGGGAGGCCCGT |  |
| MoMdv1-F3 | ACTCACGGGCCTCCCTACTCTTCGTTTAAACCAACACCATCTCTTGGACATGG |  |
| MoMdv1-F4 | TTCAAGACCCCGTACGACAACGT |  |
| MoMdv1-IF | ATACGGCAGTGGGATCTGGTCAA | Amplify *MoMDV1* gene probe, for southern blot and transformants screen |
| MoMdv1-IR | CTTACACGTTCGACAATGGCAG |  |
| MoMdv1-OF | AGACTCGTAGCGGCGAATTAGA | Transformants screen |
| MoFis1-Pro-F | ACTCACTATAGGGCGAATTGGGTACTCAAATTGGTTAAGAAGGTCGCAATACATGGCG | *MoFIS1* complementation  (MoFis1-Pro-F, MoFis1-pro-R, MoFis1-GFP-F, MoFis1-GFP-R, MoFis1-Com-F are used in construction of MoFis1^Δ127-155^ and MoFis1^Δ148-155^) |
| MoFis1-pro-R | TTTTGATGATATGCCTGATGTCTAG |  |
| MoFis1-GFP-F | TTTATATTCTAGACATCAGGCATATCATCAAAA ATGGTGAGCAAGGGCGAGGA |  |
| MoFis1-GFP-R | CTTGTACAGCTCGTCCATGC |  |
| MoFis1-Com-F | ATCACTCTCGGCATGGACGAGCTGTACAAGATGGGGACTAATCTTCCCTGTGAGT |  |
| MoFis1-Com-R | TTACTTGTACAGCTCGTCCATGCCGAGAGTGATCTACCTCTTCCTGCCCATGTTCCT |  |
| MoMdv1-Pro-F | CTATAGGGCGAATTGGGTACTCAAATTGGTT TCAGCCTGTCCAGAACTTGCTT | *MoMDV1* complementation |
| MoMdv1-Pro-R | GAAGAGTAGGGAGGCCCGT |  |
| MoMdv1-GFP-F | GGCACTCACGGGCCTCCCTACTCTTCATGGTGAGCAAGGGCGAGGA |  |
| MoMdv1-GFP-R | CTTGTACAGCTCGTCCATGC |  |
| M Mdv1-Com-F | ATCACTCTCGGCATGGACGAGCTGTACAAGATGGCGTCTCCCGAGGATGAAAAT |  |
| MoMdv1-Com-R | TTACTTGTACAGCTCGTCCATGCCGAGAGTGATTCAGCAAGTCCAAATGCCCACA |  |
| BD-MoFis1-F | TAATAACATATGATGGGGACTAATCTTCCCTATGC | Construction of *pGADT7-MoFIS1* and *pGBKT7-MoFIS1* |
| BD-MoFis1-R | TAATAAGAATTCCTACCTCTTCCTGCCCATGTT |  |
| GST-MoFis1-F | TAATAAGAATTCCCATGGGGACTAATCTTCCCTATGC | Construction of *GST-MoFIS1* |
| GST-MoFis1-R | TAATAAGCGGCCGCCTACCTCTTCCTGCCCATGTT |  |
| *HIS_6_-*-MoFis1-F | TAATAAGATATCATGGGGACTAATCTTCCCTATGC | Construction of *HIS_6_-MoFIS1* |
| *HIS_6_-*-MoFis1-R | TAATAAGAATTCCTACCTCTTCCTGCCCATGTT |  |
| AD-MoMdv1-F | TAATAACATATGATGGCGTCTCCCGAGGATGAA | Construction of *pGADT7-MoMDV1* |
| AD-MoMdv1-R | TAATAAGAATTC TCAGCAAGTCCAAATGCCCACAAT |  |
| *HIS_6_-*MoMdv1-F | TAATAAGATATCATGGCGTCTCCCGAGGATGAA | Construction of *HIS_6_-MoMDV1* |
| *HIS_6_-*-MoMdv1-R | TAATAAGAATTC TCAGCAAGTCCAAATGCCCACAAT |  |
| MoFis1^Δ1-29^-F | TTT CGT AGG AAC CCA ATC TTC AAA ATGGAGGGCGACATGGTTGGTGT | Construction of MoFis1^Δ1-29^ (MoFis1^Δ1-29^-R also used in construction of MoFis1^Δ1-57^ and MoFis1^Δ1-88^) |
| MoFis1^Δ1-29^-R | TTACTTGTACAGCTCGTCCATGCCGAGAGTGATCTACCTCTTCCTGCCCATGTTCCT |  |
| MoFis1^Δ1-57^-F | TTT CGT AGG AAC CCA ATC TTC AAA ATGGGGGTCATGCTCCTCTCCGAA | Construction of MoFis1^Δ1-57^ |
| MoFis1^Δ1-88^-F | TTT CGT AGG AAC CCA ATC TTC AAA ATG AACTACGCAGAGGCGAGACGGTA | Construction of MoFis1^Δ1-88^ |
| MoFis1^Δ127-155^-R | TTACTTGTACAGCTCGTCCATGCCGAGAGTGATTTACAGTCCCTCCTTGGCGACCTT | Construction of MoFis1^Δ127-155^ |
| MoFis1^Δ127-155^-R | TTACTTGTACAGCTCGTCCATGCCGAGAGTGAT TTAGATAAGTATTCCACCGACGA | Construction of MoFis1^Δ148-155^ |
| 28S rDNA LL | TACGAGAGGAACCGCTCATTCAGATAATTA | qRT-PCR |
| 28S rDNA RR | TCAGCAGATCGTAACGATAAAGCTACTC | qRT-PCR |
| Rubq1 LL | GTGGTGGCCAGTAAGTCCTC | qRT-PCR |
| Rubq1 RR | GGACACAATGATTAGGGATCA | qRT-PCR |
| Pex11a--F1 | GATAAGCTTGATATCGAATTCACACTACAGAGCATGCTAGT | *Amplify MoPEX11A 5’ flank sequence, for gene knock out* |
| Pex11a-R1 | ATGCTCCTTCAGGTACCCTCGTTTGGGCTATTTGGTTTGTTGTTT |  |
| Pex11a-Ble-F | AAACAACAAACCAAATAGCCCAAACGAGGGTACCTGAAGGAGCAT | *Amplify BLE sequence, for gene knock out* |
| Pex11a-Ble-R | ACCTGAAAGACCGGTTTGAGCAAGATGAGCTGTATCTGGAAG |  |
| Pex11a-F2 | CTTCCAGATACAGCTCATCTTGCTCAAACCGGTCTTTCAGGT | *Amplify MoPEX11A 3’ flank sequence, for gene knock out* |
| Pex11a-R2 | TCCACCGCGGTGGCGGCCGCTTGTGCGCGTCTAGCATCGACT |  |
| Pex11a-IF | TGTCGCACTACCTCAAGTTCGT | Amplify *MoPEX11A* gene probe, for southern blot and transformants screen |
| Pex11a-IR | TCTCACTGTAGTTGGCCACCAT |  |
| Pex11a-OF | TATGACTGGCAGCTTAGCCAGAG | Transformants screen |
| BLE ConR | TGGGCGAAGAACTCCAGCATGAGA |  |
